# Stockholm Score of Lesion Detection on Computed Tomography following Mild Traumatic Brain Injury (SELECT-TBI) Study: Pilot Analysis and Statistical Analysis Plan

**DOI:** 10.1007/s00701-025-06598-1

**Published:** 2025-07-01

**Authors:** Li Jin Yang, Charles Tatter, Alexander Fletcher-Sandersjöö, Logan Froese, Philipp Lassarén, Jonathan Tjerkaski, Erica E. Bergman, Frida E. Björkman, Jonas Bronge, Julia Antonsson, Kasper Teromaa, Maria Nylander, Simon Örtqvist, William Kylander, William Lindqvist, Kristian Ängeby, Rebecka Rubenson Wahlin, Eric P. Thelin

**Affiliations:** 1 Department of Emergency Medicine, Stockholm South General Hospital, Stockholm, Sweden; 2 Department of Clinical Science and Education Södersjukhuset, Karolinska Institutet, Stockholm, Sweden; 3 Department of Radiology, Stockholm South General Hospital, Stockholm, Sweden; 4 Department of Clinical Neuroscience, Karolinska Institutet, Stockholm, Sweden; 5 Department of Neurosurgery, Karolinska University Hospital, Stockholm, Sweden; 6 Department of Perioperative Medicine and Intensive Care, Karolinska University Hospital, Stockholm, Sweden; 7 Medical Unit Neurology, Karolinska University Hospital, Stockholm, Sweden

**Keywords:** Statistical analysis plan, Traumatic brain injury, Head injury, Intracranial lesion, Neurosurgery

## Abstract

**Background:**

Mild traumatic brain injury (mTBI) is a common cause of emergency department visits. Only a small percentage of mTBI patients develop an intracranial lesion (ICL) and even fewer will require neurosurgical intervention due to their injury. The Stockholm Score of Lesion Detection on Computed Tomography following Mild Traumatic Brain Injury (SELECT-TBI) study aims to provide a data-driven approach to estimate individualized risk for traumatic ICL and clinically significant lesions in mTBI patients.

**Objective:**

To provide a statistical analysis plan and pilot data analysis before completion of data collection, as pre-planned in the published study protocol.

**Methods:**

Retrospective study of patients ≥ 15 years old who underwent a computed tomography (CT) scan for their mTBI in Stockholm, Sweden, between 2015–2020. Up to 73 variables were collected for each patient. Data analysis of the first 5 000 patients in the cohort was conducted to develop preliminary prediction models using Lasso regression, general linear model and random forest and to perform an optimal population analysis to determine whether the final sample size would be sufficient.

**Results:**

Six data selection strategies were tested, and area under the curve (AUC) receiver operator characteristic (ROC) curves were generated with a 4:1 training/validation data segmentation. The best-performing model was the Lasso regression model which achieved an AUC of 0.807 for any ICL and 0.903 for clinically significant ICL (accuracy of 70% and 97.7%, and Brier scores of 0.3 and 0.023 respectively). Clinical variables identified as key features across all models were Glasgow Coma Scale, signs of basilar skull fracture, trauma mechanism, and vomiting, each with an importance score greater than 0.1 (explaining more than 10% model variance). Finally, the highest end prediction of the necessary population size was found to be 29 667 patients.

**Conclusion:**

Our preliminary results demonstrate the potential for a data-driven approach to generate personalized risk stratification tools. With a final cohort size expected to exceed 40 000 patients, we anticipate being able to create more granular models optimized for integration into clinical decision-making.

**Study registration:**

ClinicalTrials.gov NCT04995068.

**Supplementary Information:**

The online version contains supplementary material available at 10.1007/s00701-025-06598-1.

## Introduction

Traumatic brain injury (TBI) is one of the leading causes of morbidity and mortality worldwide and one of the main causes of emergency department (ED) visits, with more than 60 million cases annually [5]. The Glasgow Coma Scale (GCS) is currently the most widely applied method for the classification of TBI and has been shown to be predictive of outcome [28, 29, 31]. Mild traumatic brain injury (mTBI), defined as a head injury patient with a GCS score of 13 to 15, accounts for an estimated 70–90% of all TBI cases [4]. Previous studies have shown that only a small portion of these patients will develop an intracranial lesion (ICL) visible on a head computed tomography (CT) scan [22, 27] and less than 1% will require neurosurgical intervention [6].

The detection of an ICL has important clinical consequences, such as the potential need for hospitalization for observation, reversal of anticoagulation and repeat head CT scans. A subsequent clinical deterioration leading to surgical intervention is a more severe outcome where early intervention is critical to patient survival. Although numerous guidelines exist for the clinical management of patients with mTBI in the ED setting [12, 15, 18, 26, 27, 34], most focus primarily on selecting patients for CT imaging and admission. Several studies have also investigated predictors of neurosurgical intervention [21, 23, 32], but there is a need for more precise characterization of these predictors and adjustment for risk factor interactions.

The ongoing Stockholm Score of Lesion Detection on Computed Tomography following Mild Traumatic Brain Injury (SELECT-TBI) study aims to develop one of the largest and most comprehensive multicenter retrospective cohorts of adults with mTBI, encompassing over 40 000 unique patient profiles. The objective is to create models for individualized risk estimation for a traumatic ICL as well as clinically significant ICLs (defined as those requiring neurosurgical intervention, intubation or resulting in death) in mTBI patients who present to the ED. As outlined in our published study plan [7], we will describe the pre-planned statistical analysis and produce initial models using pilot data before the completion of data collection and finalization of the dataset. This will include a preliminary population analysis to ensure that our expected population size will adequately contain the more unusual potential risk factors (such as dual antiplatelet therapy (DAPT) and ventriculoperitoneal (VP) shunts). Some elements in this statistical analysis plan were previously published as a part of the study plan. This preliminary work will better inform our final analysis, highlighting the potential pitfalls and documenting the strengths.

## Methods and analysis

### Study setting

This pilot analysis is conducted on the first 5 000 patients collected in our retrospective cohort from the SELECT-TBI study on adults with mTBI in the ED setting. Variables that are potential predictors of ICL were collected from the patient history, physical examination and laboratory results. The Transparent Reporting of a Multivariable Prediction Model for Individual Prognosis or Diagnosis (TRIPOD) guidelines [33] were adhered to, and the study is registered at ClinicalTrials.gov (NCT04995068).

### Study population

This pilot analysis included 5 000 patients who sought ED care for a mTBI at the Karolinska University Hospital Huddinge, Karolinska University Hospital Solna or Stockholm South General Hospital. All hospitals share the same prehospital TBI management protocol [2], and adhere to the Scandinavian Neurotrauma Committee (SNC) guidelines for initial management of minimal, mild and moderate head injuries [34]. The Karolinska University Hospital Solna and Stockholm South General Hospital also have the capabilities to sample the brain biomarker S100B, included in the SNC guidelines to rule out the need for a CT scan. Patients were adults (≥ 15 years) with mTBI (GCS 13–15) who presented to the ED within 24 h of injury between 2015 and 2020 (Table 1), with patient records used to collect the predefined clinically relevant variables. All included patients have undergone a head CT. Patients have been identified by a systemwide search in the electronic medical records software for International Classification of Diseases, Tenth Revision (ICD-10) codes for intracranial injury (S06X) and fracture of skull and facial bones (S02X), as well as the ED admissions codes for ‘head injury’.

**Table 1:** Data used to create the key models. All models used the same initial population size of 5 000 patients, with patients excluded if there were missing data points. Exclusion of variables (columns) leads to more patients being included in the models. Model A retains the most patients with the most columns removed and Model F inversely has the most variables, but the most patients removed due to missing data. INR = international normalized ratio, APTT = activated partial thromboplastin time

Models	Patient Count	Variables (Only Patients with Complete Data Included)
A	4 668	Hemoglobin, platelet count, S100B, INR, APTT and time to admission removed
B	3 465	Hemoglobin and platelet count included, S100B, INR, APTT and time to admission removed
C	656	Hemoglobin, platelet count and S100B included, INR, APTT and time to admission removed
D	1 534	Hemoglobin, platelet count, INR and APTT included, S100B and time to admission removed
E	2 142	Hemoglobin, platelet count and time to admission included, S100B, INR and APTT removed
F	106	Hemoglobin, platelet count, S100B, INR, APTT and time to admission included

### Data collection

Clinical variables were retrospectively collected using a standardized review protocol and conducted by members of the SELECT-TBI study group. Clinical data was collected from the health record software TakeCare (CompuGroup Medical Sweden AB, Farsta, Sweden), while imaging data was collected from the radiological management software Sectra Picture Archiving and Communication System (Sectra AB, Linköping, Sweden). The data was entered into anonymized case report forms in the electronic data capture system REDCap [11]. The primary outcome was any traumatic ICL on head CT, defined as an intracerebral hematoma, subdural hematoma, epidural hematoma, subarachnoid hemorrhage, intraventricular hemorrhage, diffuse axonal injury, depressed skull fracture, traumatic infarction or sinus thrombosis. The secondary outcome was any clinically significant lesion, defined as a traumatic ICL that led to neurosurgical intervention, intubation, or death. In a sub-analysis, we also removed fractures, specifically looking at outcome of patients with intracranial hematomas. Due to the retrospective nature of the study, each patient has already been assessed at their index ED visit by a physician who determined the need for a head CT with support from regional guidelines [34], and the results of the CT scans have been interpreted by site faculty radiologists (including a board certified radiologist). The variables have been chosen due to their previously suggested relationship to intracranial lesion risk in mTBI decision rules, namely the Canadian CT Head Rule [27], New Orleans Charity Head Trauma Rule [12], National Institute of Healthcare Excellence guidelines [14], CT in Head Injury Patients Prediction Rule [26], NEXUS II Head CT Decision Instrument [20], SNC guidelines [34], Brain Injury Guidelines [15], the Mild TBI Risk Score [18], as well as other risk factors investigated in previous literature. The complete variable list is presented in the appendix of our study plan [7].

### Data definitions

A few key definitions of the variables included in SELECT-TBI require clarification.

GCS: Previous studies in the ED setting show a moderate level of interrater reliability with GCS [8, 9]. The first documented physician assessment of GCS was used in our study as the standard value. Where only descriptions of levels of consciousness were available, a best approximation was made through consensus within the record review group.

Medication: Antiplatelet and anticoagulant therapies were documented when the patient was determined to be on the specified prescription and compliant to the treatment. If the patient was found to have not taken the prescription > 24 h from the time of trauma, the therapy is considered ineffective (or in the case of warfarin, a normalization of INR), and the medication is disregarded.

Liver failure and renal failure: These diagnoses are based on information available during the timeframe for decision-making for the CT order and are considered a comorbidity if the clinician observed and documented these conditions in the patient history and not based on lab findings.

Intoxication: Based on pre-CT clinician assessment and documentation, which may or may not have included breathalyzer (alcohol meter) or blood laboratory results.

Scalp wound: A wound requiring treatment (sutures or glue) above the eyebrow level, reflecting a likely direct injury to the neurocranium.

Multitrauma: Defined as a suspected injury prompting the physician to order additional imaging in addition to head and neck CT (i.e. not the result of the CT, as the SELECT-TBI score is designed to work prior to the CT being performed).

Trauma mechanism: To compare the significance of trauma energy level, trauma mechanisms were classified according to the Utstein Trauma Template for Uniform Reporting of Data following Major Trauma [25], with high energy defined as high-level fall (> 1 m or > 5 steps), struck by blunt object, motor vehicle accident (not motorcycle), motorcycle accident, bicycle accident, pedestrian hit by traffic, other traffic accident, and blast injury (e.g. explosion), and low-energy defined as low-level fall (same level). Trauma mechanisms not meeting this classification were excluded from the final analyses.

Dementia/cognitive impairment: Any documented history of known chronic cognitive impairment, specific diagnosis not required.

Laboratory data: Furthermore, as this was a retrospective study using clinical data, some variables have clinically relevant thresholds for the adjustment of care. This includes INR > 1.1, APTT > 32, S100B > 0.1 µg/L, Hemoglobin < 120 g/L and Platelets > 150 × 10^9^/L [17]. We have thus added 5 extra variables for these categories, where the value above and below the threshold was marked as 1 and 0, respectively.

### Statistics

The statistical analysis in this paper is conducted on the first 5 000 patients included in the SELECT-TBI study. We also performed a complete analysis of the population size to help us better estimate how many patients are needed to adequately assess the clinical impact of presumed risk factors that are rarer (e.g., DAPT and VP shunts). This work leveraged the manuscript from Riley et al. [24], on the minimum sample size required for a clinical prediction model to estimate the overall outcome proportion with sufficient precision, and to target the magnitude of required shrinkage of predictor effects (to minimize potential model overfitting) for binary outcomes, with more details found in the referenced literature including method equations for overall outcome proportions and shrinkage predictor effects. Given the nature of this analysis, we will only demonstrate the values for our analysis that maximize the patient population requirement. This work can be repeated for all outcomes/variables, but the overall minimum required patient population size will remain the same.

To complete the initial modeling of the data we leveraged a general linear model (GLM), random forest (RF) and Lasso regularized regression (LR) approach with cross-validation [1, 16, 30]. We segmented the data into 80% training and 20% testing. We tested 6 different patient sets with the 3 different modeling methods, resulting in 18 different models tested, with a feature model highlighted in the main article while additional models shown in the Appendix. Table 1 displays the specific variable selection for each model population. For the input data, we ensured that each variable analysis had at least 50 patients within the cohort (1% of total population size). The models were chosen based on what are likely different clinical settings worldwide (a low resource availability scenario with only clinical assessments, and a high resource availability scenario with access to blood tests for coagulation assessment and usage of the TBI biomarker S100B). Each model was also based on the initial data, with subsequent segmentation based on model requirements and missing data. Thus, no imputation on the data has yet to be performed. Area under the receiver operating characteristic curve (AUC) was used to assess model performance, with overall model variance for each variable used to define feature importance. Overfitting was avoided by using regularized regression, which will eliminate poorly performing predictors. Furthermore, the validation set will give a non-biased estimate of the performance of the final score internally.

Brier score was also found for each model, and calibration analyses performed for each population assessed. Multicollinearity for GLM was found through variance inflation factor (VIF), with LR implicitly accounting for this by shrinking multicollinear variables and RF making low importance and highly correlated variables redundant [3, 19].

### Treatment of missing data

Due to the preliminary nature of this manuscript, there were no efforts made to preserve patients to whom data was missing in certain categories or to perform imputations. Moreover, the percentage of the population removed from each analysis will likely be similar to that of the finalized larger analysis.

### Patient and public involvement statement

Patients or the public were not involved in the design, or conduct, or reporting, or dissemination plans of our research.

## Results

### Patient demographics

Summarizations for each build model can be found in Appendix Tables A1 to A6. Table 2 demonstrates the data for the full population analyzed. Of the 5 000 patients, the median age was 69 years (interquartile range; IQR: 47–83 years), with 2 577 (51.5%) being males.

**Table 2:** Patient demographics for all data. The demographics for the initial 5 000 patients used in the pilot analyses. Data presented as count (proportion) or median (interquartile range)

Variable	All Patients (*n* = 5 000)
Number of Patients	5 000
Sex (Percentage Male)	2 577 (51.5%)
Age (Years, Median with Interquartile Range)	69 (47–83)
GCS 13	124 (2.4%)
GCS 14	739 (14.8%)
GCS 15	4 137 (82.7%)
Primary Outcome	666 (13.3%)
Secondary Outcome	51 (1.1%)

### Recommended population size for final analysis

The steps required for this analysis are describe in great detail within the work by Riley et al. [24], though as a summary the primary outcome (portion = 0.13, 666 patients) and VP-shunts (portion = 0.006, 30 patients) were used, as they necessitate the largest sample size. To ensure a 95% confidence interval for the outcomes, an overall sample size of 178 patients is required. Even in the most extreme scenario – considering 178 patients per variable (totaling 12 994 patients for 73 variables) or using the most infrequent variable (VP shunts) with 29 667 patients (178 patients/0.006, the prevalence of VP shunts in the pilot population) – the projected cohort size of > 40 000 patients is sufficient.

For the assessment of the shrinkage aspect for the variables (limiting overfitting), using the maximal R^2^ value of 0.3 (found below) indicates a population requirement of 18 507 patients, again well below our expected final cohort population.

### Highlighted features

**Fig. 1:**
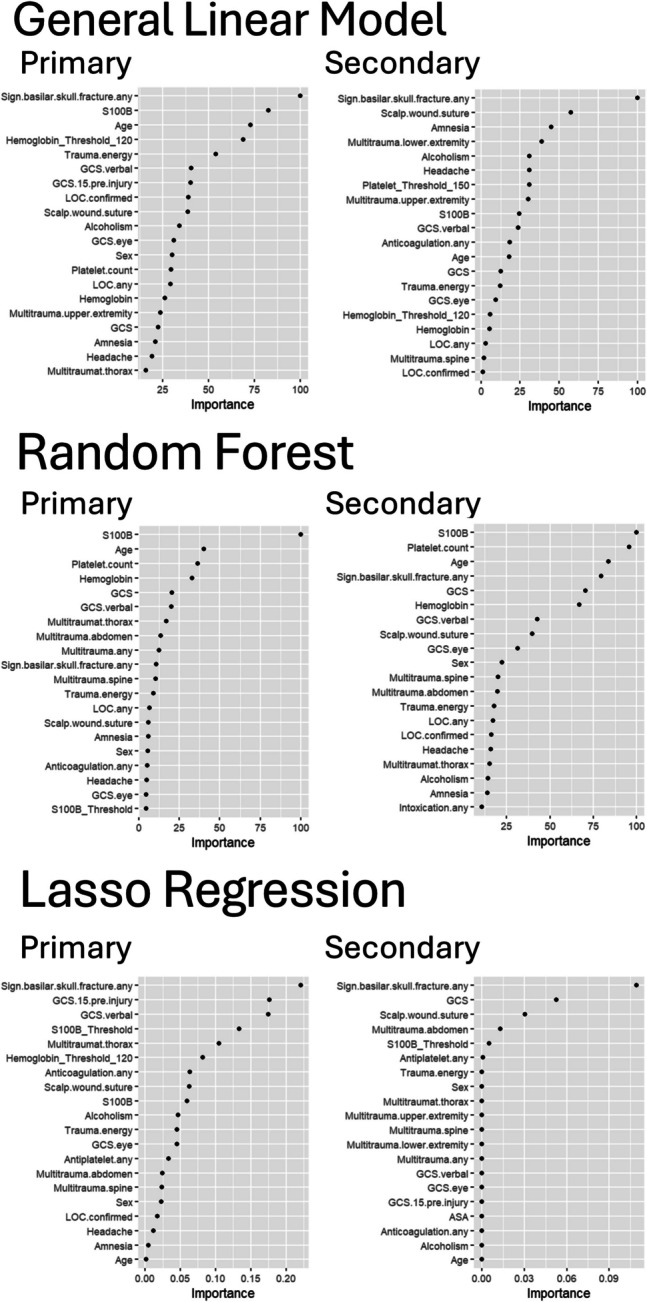
Top 20 features from model C. The top 20 most important features for model C, based on both the primary and secondary outcomes, applying general linear model, random forest and Lasso regression. Feature importance is assessed by the variance explained by each variable within the overall model, with higher variance indicating a greater contribution to the outcome. GCS = Glasgow coma scale, DOAC = direct oral anticoagulation, LOC = loss of consciousness

Figure 1 (from model C, Complete Data + Hemoglobin/Platelet Count + S100B) and Appendix B/D (all other models) demonstrate the 20 most important features for each model (as described by the amount of variance within the model captured by each feature). This work consistently demonstrated that the features of GCS deterioration, signs of basilar skull fracture, GCS, trauma energy and vomiting were the most significant throughout the various models. For most models the top 6 features presented with the most variance (> 0.2) and was limited after that. Intracranial hematoma as an outcome can be found in Appendix D, with similar overall outcome variables; with the added variable of S100B > 0.1 µg/L having a more significant role in both Model C and F (models with S100B). Finally, as highlighted by the random forest method, as a stand alone variable S100B was most strongly associated with the outcome.

### Model accuracy

**Fig. 2:**
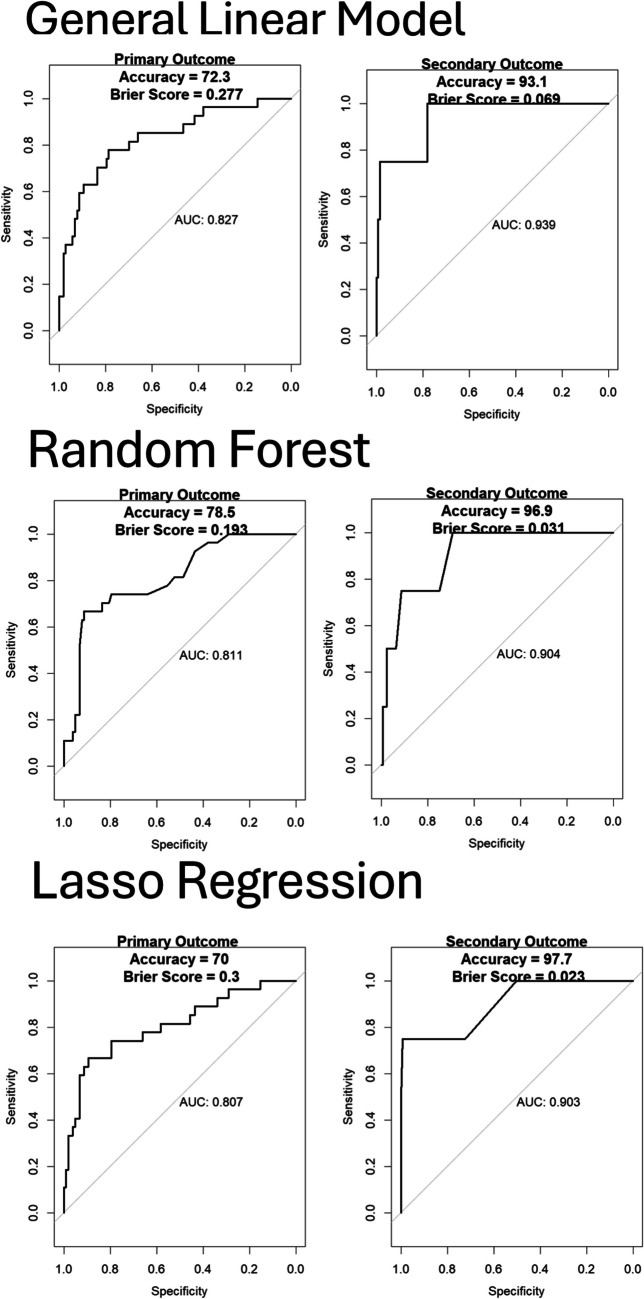
ROC curves for model C, The ROC curves for the primary and secondary outcomes were generated using the training data to create the model and testing data to evaluate the final performance of the general linear model, random forest and Lasso regression models. AUC = area under the curve, ROC = receive operation curve

Figure 2 (Model C) and Appendix C/D (all other Models) demonstrate the ROC curves for the described data. The AUC is generated from the segmented test data (20% of the population), with the models created from the training data (80% of the population). Model C (where hemoglobin, platelet count and S100B were included) had the best overall AUC, accuracy and Brier score to describe both the primary and secondary outcomes. Of these, the LR model had the best results with AUC of 0.807 and 0.903, accuracy of 70% and 97.7% and a Brier score of 0.3 and 0.023, for the primary and secondary outcomes respectively. Comparing these results with Model D (see appendix, figure C3, page 23, hemoglobin, platelet count, INR and APTT included), which had an AUC value 0.721 and 0.861, indicates that inclusion of some of these lab values as linear or threshold-based variables will have an effect on the predictive ability of the final model. However, this will require further investigation with the increased sample size of the final patient cohort.

### Calibration data

Appendix E demonstrates the calibration curves for each model and patient population. Given the nature of the outcomes we are assessing, in general the LR had the best overall calibration result with RF overestimating the results. However more patients are required to fully justify these results.

## Discussion and future directions

This statistical analysis plan (SAP) is published with the intent of minimizing data-driven outcome reporting bias, ensuring the final population size is adequate and highlights some risk factors with predictive ability for mTBI outcome based on our preliminary data from the SELECT-TBI cohort. Based on the prevalence of variables in our pilot analysis population, we have demonstrated that our expected final population size of 40 000 patients meets the sample size requirement to ensure model accuracy. The GLM, RF and LR models we applied was initially chosen for there relatively simple implementation and its ability to select variables through shrinkage while limiting overfitting [30]. However, to better explore the relationships between our risk factors and outcomes, for our finalized presentation of the data we will apply other modeling methodologies in parallel with our presented method to provide alternative descriptions of the non-linear relationships and develop more robust models. Such work will leverage decision tree selection of key features, as well as methodologies like “inverse supervised learning” with neural networking, seeking to find normalized models for healthy patients [13]. We also plan to include an in-depth exploration into patient outliers (incorrectly labelled patients) as they will better describe how the model needs to be adapted for overall patient improvement.

Our aim is to apply the findings from our variable selection testing in models A to F to optimize the variable selection in our final SELECT-TBI risk stratification scores to provide two sets of models. The primary base model will utilizing universally available variables, with no loss of sample size, while the secondary SELECT-TBI plus model will add an optimized selection of ordinal variables, providing improved model performance at the cost of a lower available sample size. This approach is a reflection of clinical realities globally where clinical variables from the base model are likely to be universally available, but additional ordinal variables such as hemoglobin and platelet count are limited in availability and even fewer centers have the possible of utilizing routine testing of mTBI patients with specific biomarkers such as S100B.

Applying this variable selection strategy, after the ongoing data collection is completed, we will make more poignant comments surrounding variable accuracy for the final models. We will apply LR and RF methodology to highlight key features which preliminarily demonstrated an approximate cutoff around 0.8 AUC. In addition to this, other methods such as a neural networking to assess the importance of all defined variables will also be explored. In this way we will be able to assess the importance of each feature in the overall model performance with greater detail.

From our analysis, some key predictors were identified in the preliminary models (GCS deterioration, signs of basilar skull fracture, GCS, trauma energy and vomiting) that may require closer examination as they consistently demonstrated importance in the models. As a stand alone variable S100B was highlighted as the most important variables as demonstrated by the RF method. With the exception of trauma energy level, these features are variables that are currently applied in some but not all clinical guidelines [12, 26, 27, 34] as well as emerged as significant predictors of ICL in a recent systematic review [35] which supports the external validity of the elements in our preliminary model. Future iterations of the final model will be tailored to the clinical context, necessitating a pragmatic reduction in the number of variables. Variable selection will prioritize those with sufficient incidence in the mTBI population to ensure utility for rapid decision-making in the ED setting. A concrete example of a clinical challenge we hope to be able to address using our final models are the confounding effects from intoxication, dementia and cognitive impairment on decreased level of consciousness during ED assessment. Moreover, these situations will also result in affected self-reporting about the trauma, with an increased risk of CT overuse. Similarly, there is also a risk for CT overuse in the case of anticoagulants, as all patients independent of TBI severity (even minimal TBI), should have a CT scan performed if taking anticoagulants according to the SNC mTBI guidelines [34]. These aspects need to be taken into consideration in the final prediction models. In all, we aim to create and improve models for ICL prediction and provide a more robust statistical exploration into interactions between patient physiological factors that dictate mTBI outcomes.

There is some variability in the definition of ICL in mTBI literature, with some studies choosing to include neurocranial and basilar skull fractures and others only including hemorrhagic lesions. In order to account for this variation, we have included a secondary analysis of only hemorrhagic lesions in our appendix, demonstrating that there seem to be few differences in the key features of the models between these two definitions of ICL. Interestingly, our preliminary models seem to perform somewhat better for the secondary outcome of clinically significant lesions when compared to all ICLs, with an AUC range of 0.5–0.903 in comparison to 0.5–0.807. We see this as a promising result as we consider predictors and predictive models of clinically significant lesions a key knowledge gap that we would like to explore in more detail in the final cohort analysis. However, the highest AUC values from the secondary models were a consequence of low prevalence of the secondary outcome in the trimmed model population sample that is further reduced in the validation set. We do not expect the final model with a fully adequate sample size to have unnaturally high AUC values. One limitation with the outcome “death due to TBI” is that we have not thoroughly assessed if TBI was the cause of death but rather whether the patient died during their hospital care. While only a few patients are likely to have died from hematoma progression and cerebral herniation, it can be very challenging to determine the contribution of mTBI to the cause of death, as many patients are old with comorbidities. For example, an elderly woman with hip problems may become more immobilized due to the mTBI and then pass away due to pneumonia or a pulmonary embolism. Additionally, in elderly and critically ill patients, there is a risk that treatment is withheld, which could affect this outcome. Careful consideration and detailed hospital chart reviews will be conducted for the final model.

As highlighted by a systemic review on clinical decision rules for mTBI, most existing decision models have a sensitivity of 99–100% with a specificity of 48–77% [10]. Of these models, most used a fixed high sensitivity (usually at 100%), this is due to the serious consequences of false negatives (i.e. missing patients with ICLs) and thus should be highly inclusive to all patients with this issue. This means that past models have a similar overall AUC of around 0.8 (with an accuracy of around 85%). However, these models focus primarily on CT decision rules for mTBI and in particular did not implement the use of biomarkers such as S100B which is available in our dataset. Therefore, we aim to produce finalized models with improved performance over past works, in particular providing a higher level of specificity while maintaining sensitivity. As previously stated in our study plan, we recognize that patients that have not undergone a CT is not included in our study population, and thus we cannot fully control for missed subclinical tICHs. We will screen for excluded patients in the Swedish National Patient Register for intracranial lesions within 30 days from their index ED visit to see whether there is significant impact on the sensitivity for lesion detection due to these excluded patients.

Finally, given the nature of the population some variables were not collected in the patient resulting in a significant drop of population size (from 5 000 do to as low as 150). Apart from affect model performance and results effectiveness, data collection limitations effects common applications of machine learning in a clinical environment. Our work hopes to better highlight which variables are the most important in assessing patient outcome, highlighting that future patients need this data collected. Moreover, with the final data we will also assess the overall performance of the more rarely collected variables; with a final patient population of 40 000 we expect to receive a population of about 1 200 for each group. This will allow us to sufficiently comment on the impact of these more unique collected variables to help us further optimize our final models.

## Conclusion

To our knowledge, SELECT-TBI will be one of the largest modern multicenter retrospective mTBI cohorts and the largest dataset to be applied to the development of individualized risk estimation in acute stage mTBI. This pilot SAP demonstrates the potential of Lasso regression model based on a fraction of our final dataset and provides transparency in our methodology. Moreover, with the final data, we can better highlight the most important features; making recommendation to collect vital parameters and address potential data limitation in the future. The final results of this study will provide an update to our understanding of risk interactions in mTBI and is likely to impact future guidelines.

## Supplementary Information

Below is the link to the electronic supplementary material.

Supplementary Material 1 (PDF 4.07 MB)

## Data Availability

The datasets used during the study will be available upon reasonable request.

## Abbreviations

*TBI*: Traumatic Brain Injury
*ED*: Emergency Department
*GCS*: Glasgow Coma Scale
*mTBI*: Mild Traumatic Brain Injury
*CT*: Computed Tomography
*SELECT-TBI *: Stockholm Score of Lesion Detection on CT Following mTBI
*ICL*: Intracranial Lesion
*SNC*: Scandinavian Neurotrauma Committee
*GLM*: General Linear Model
*RF*: Random Forest
*LR*: Lasso Regularized Regression
*VIF*: Variance Inflation Factor
*DAPT*: Dual Antiplatelet Therapy
*VP*: Ventriculoperitoneal
*AUC*: Area Under the Curve
*ROC*: Receiver Operating Characteristic
