# Supplementary material for: Stockholm Score of Lesion Detection on Computed Tomography following Mild Traumatic Brain Injury (SELECT-TBI) Study: Pilot Analysis and Statistical Analysis Plan
Source: Acta Neurochir (Wien). 2025 Jul 1;167(1):181. doi: 10.1007/s00701-025-06598-1 (PMC12213853; doi:10.1007/s00701-025-06598-1)
Supplement: Supplementary file 1 — Supplementary Material 1 (PDF 4.07 MB) [file 701_2025_6598_MOESM1_ESM.pdf]

1 Contents

1 Contents 1

Appendix A: Demographics 3

Appendix B: Model Features 8

Appendix C: AUC ROC Curves 18

Appendix D: Outcome Intracranial Hemorrhage 28

Appendix E: Calibration Curves 40

Appendix F: VIF for GLM 43

Appendix A: Demographics

Table A1. Patient Demographics for Model A

Variable	Number of Patients/Median (Interquartile Range)
Number of Patients	4 670
Sex (Male)	2 359 (50.5%)
Age (Years)	70 (49-83)
Primary Outcome	612 (13.1%)
Secondary Outcome	51 (1.06%)
GCS 13	96 (2.1%)
GCS 14	613 (13.1%)
GCS 15	3 959 (84.8%)

The demographics for 4 668 patients of all major complete data.

Table A2. Patient Demographics for Model B

Variable	Number of Patients/Median (Interquartile Range)
Number of Patients	3 465
Sex (Male)	1 775 (51.2%)
Age (Years)	72 (54-85)
Primary Outcome	548 (15.8%)
Secondary Outcome	46 (1.3%)
GCS 13	87 (2.5%)
GCS 14	537 (15.5%)
GCS 15	2 841 (82%)

The demographics for 3465 patients of complete data of hemoglobin and platelet count.

Table A3. Patient Demographics for Model C

Variable	Number of Patients/Median (Interquartile Range)
Number of Patients	656
Sex (Male)	396 (60.4%)
Age (Years)	54 (31-72)
Primary Outcome	137 (20.9%)

Secondary Outcome	20 (3.0%)
GCS 13	27 (4.1%)
GCS 14	120 (18.3%)
GCS 15	509 (77.6%)

The demographics for 656 patients for complete data of S100, hemoglobin and platelet count.

Table A4. Patient Demographics for Model D

Variable	Number of Patients/Median (Interquartile Range)
Number of Patients	1 534
Sex (Male)	807 (52.6%)
Age (Years)	73 (55-85)
Primary Outcome	355 (23.1%)
Secondary Outcome	35 (2.3%)
GCS 13	51 (3.3%)
GCS 14	297 (19.3%)
GCS 15	1 186 (77.3%)

The demographics for 1534 patients for complete data of INR, APTT, hemoglobin and platelet count.

Table A5. Patient Demographics for Model E

Variable	Number of Patients/Median (Interquartile Range)
Number of Patients	2 142
Sex (Male)	1 055 (49.3%)
Age (Years)	69 (46-83)
Primary Outcome	238 (11.1%)
Secondary Outcome	16 (1.8%)
GCS 13	17 (1.9%)
GCS 14	136 (15.7%)
GCS 15	713 (82.3%)

The demographics for initial 2142 patients for complete data of time to admission, hemoglobin and platelet count.

Table A6. Patient Demographics for Model F

Variable	Number of Patients/Median (Interquartile Range)
Number of Patients	106
Sex (Male)	65 (61.3%)
Age (Years)	65 (44.2-77.8)
Primary Outcome	26 (24.5%)
Secondary Outcome	4 (4%)
GCS 13	6 (5.7%)
GCS 14	25 (23.5%)
GCS 15	75 (70%)

The demographics for 106 patients for complete data of Time to admission, S100B, INR, APTT, hemoglobin and platelet count.

Appendix B: Model Features

Figure B1. Top 20 Features from Model A

General Linear Models (Primary and Secondary)

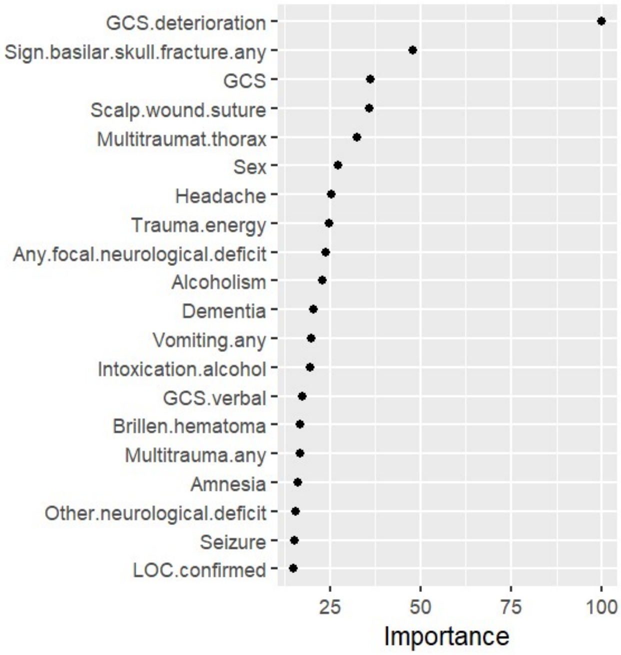

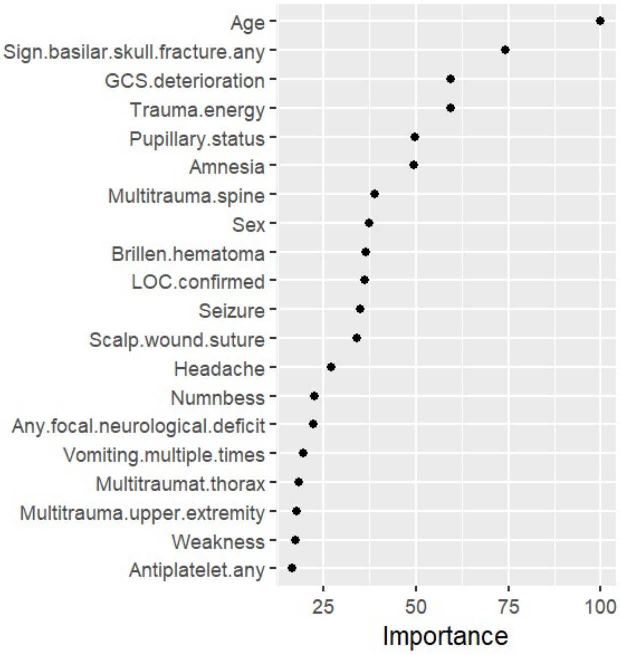

Random Forest (Primary and Secondary)

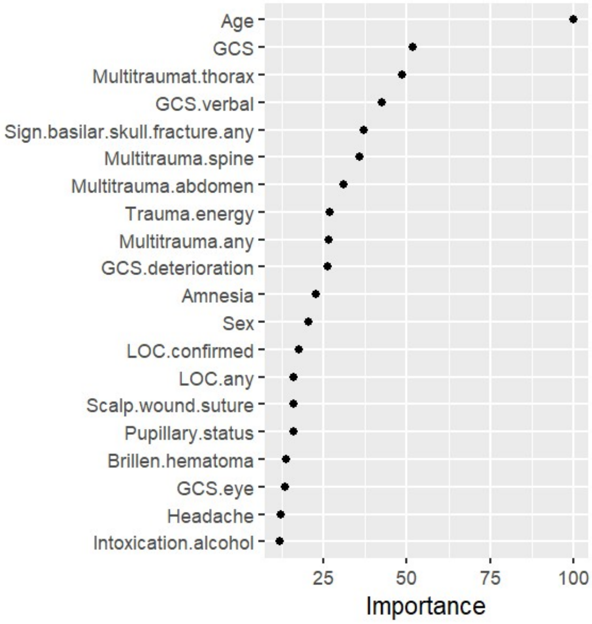

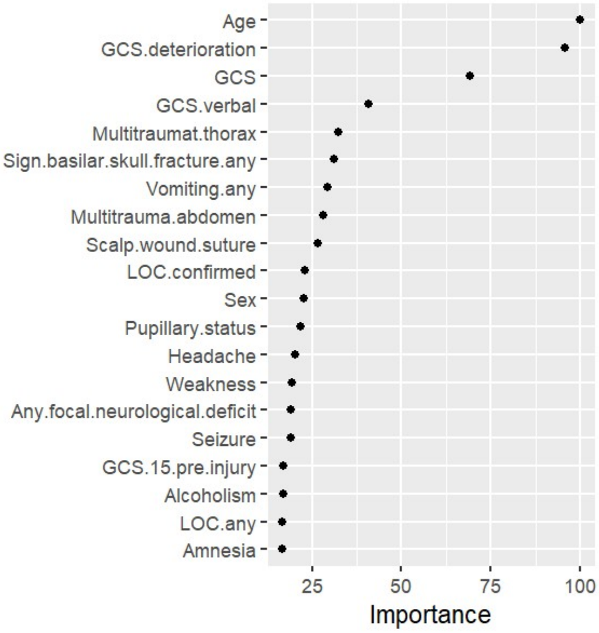

Lasso Regression (Primary and Secondary)

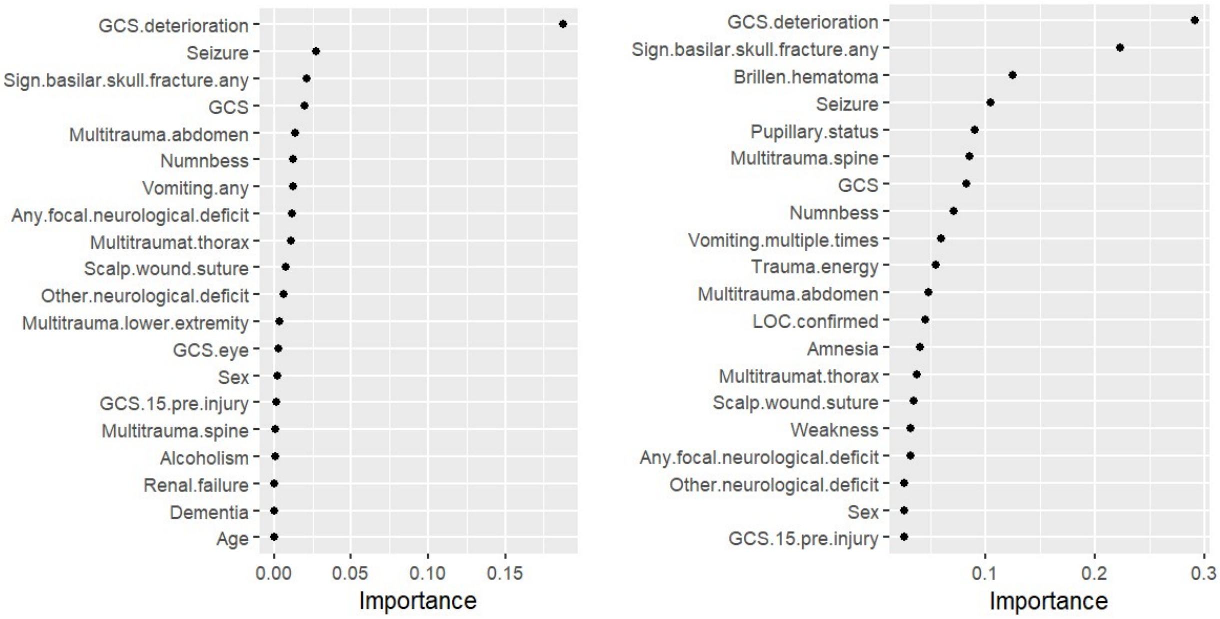

These images demonstrate the topmost 20 important features for Model A on both the Primary (left) and Secondary (right) outcomes. The feature importances uses the variance of the overall model described through each variable, noting that the variable to whom the most variance is ascribed to has the most descriptive capacity of the outcome. GCS = Glasgow Coma Scale, LOC = location, S100B = blood biomarker.

Figure B2. Top 20 Features from Model B

General Linear Models (Primary and Secondary)

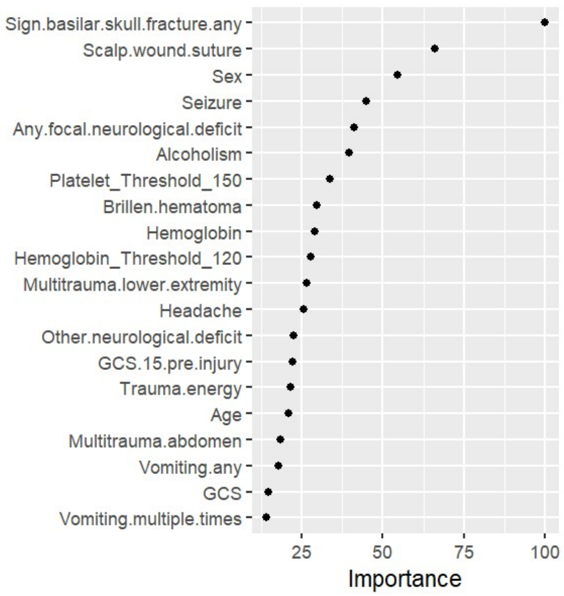

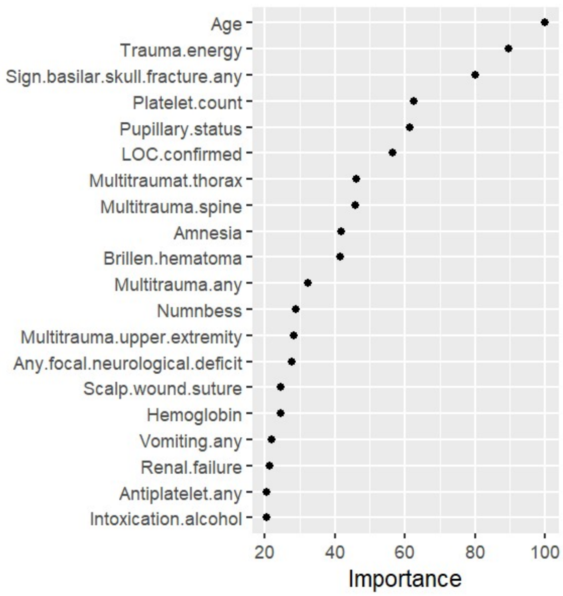

Random Forest (Primary and Secondary)

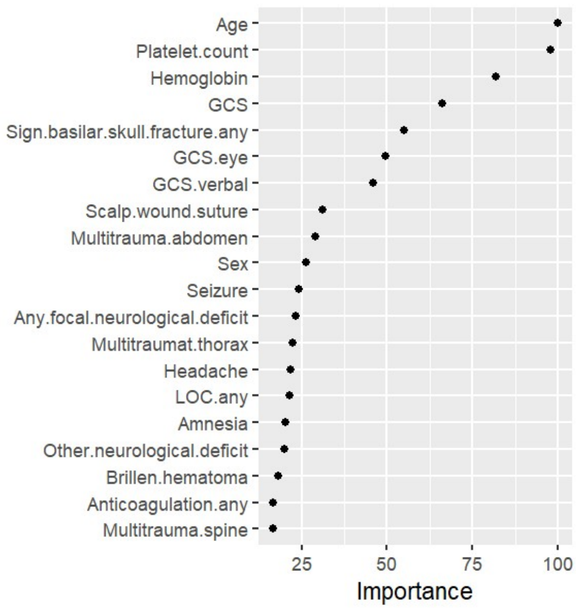

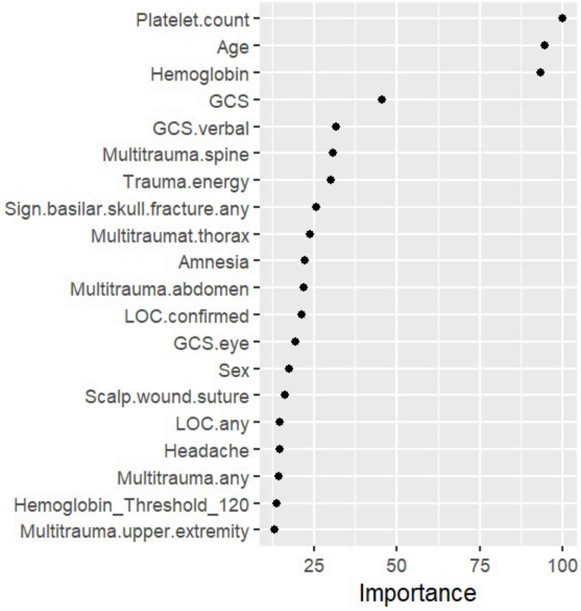

Lasso Regression (Primary and Secondary)

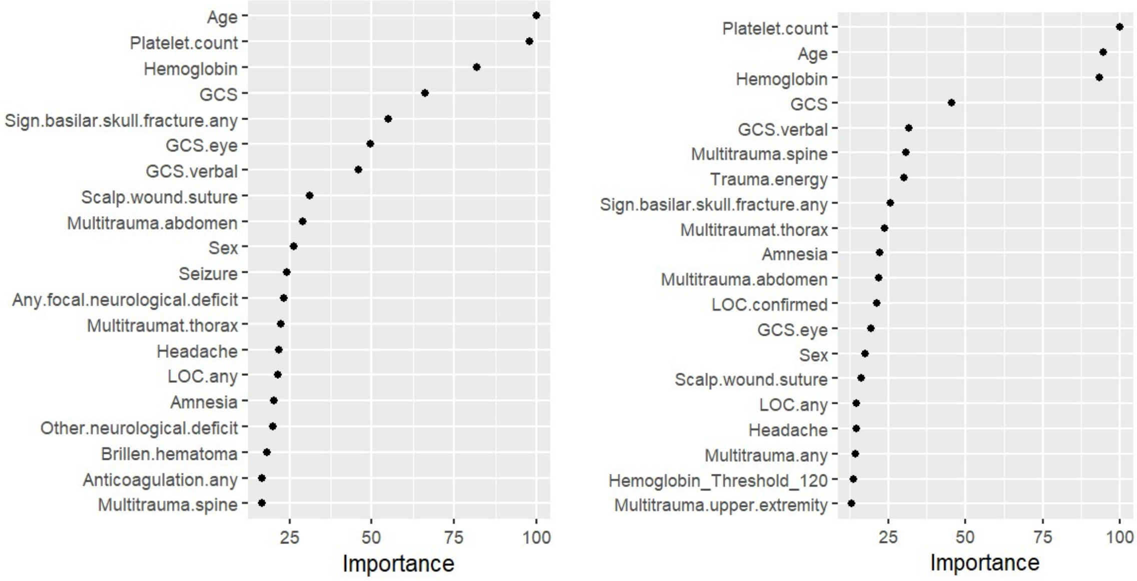

These images demonstrate the topmost 20 important features for Model B on both the Primary (Left) and Secondary (Right) outcomes. The feature importances uses the variance of the overall model described through each variable, noting that the variable to whom the most variance is ascribed to has the most descriptive capacity of the outcome. GCS = Glasgow Coma Scale, LOC = location, S100B = blood biomarker.

Figure B3. Top 20 Features from Model D

General Linear Models (Primary and Secondary)

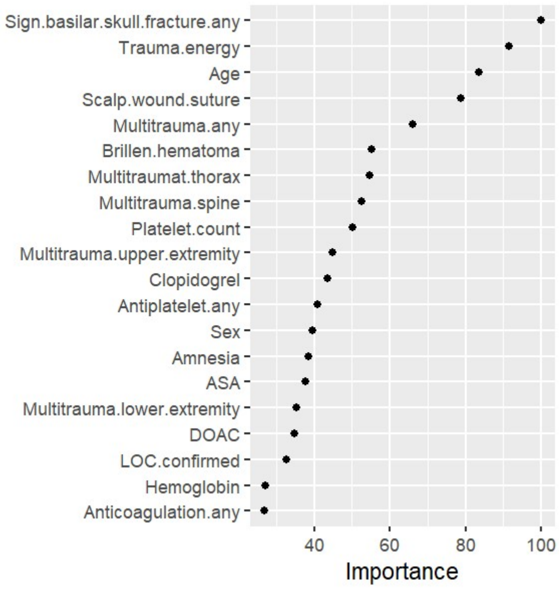

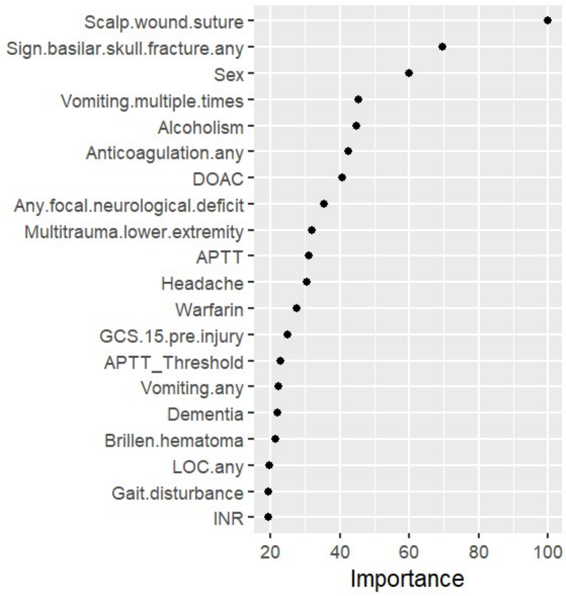

Random Forest (Primary and Secondary)

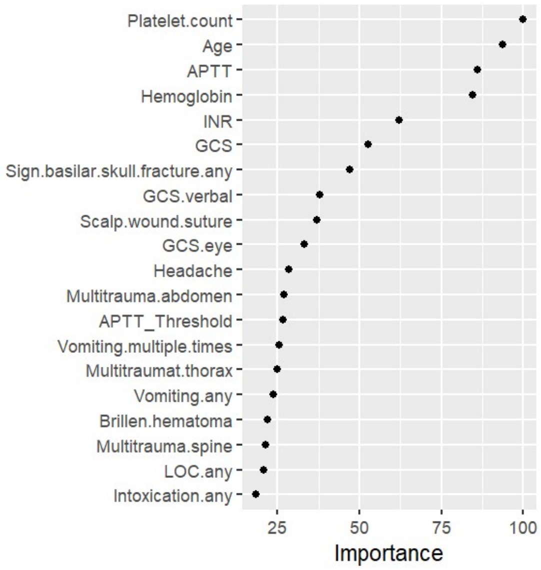

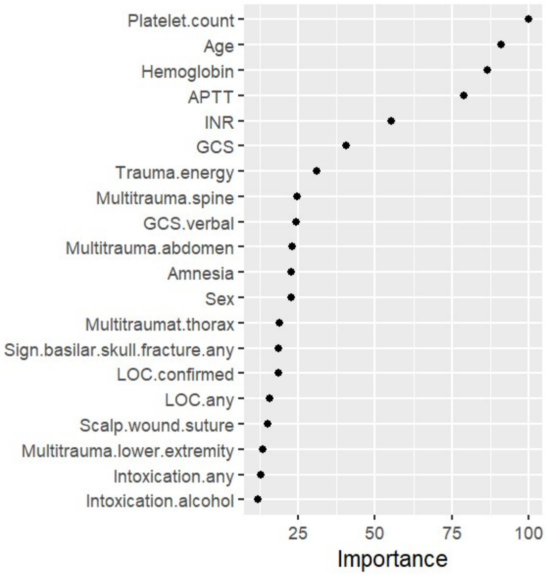

Lasso Regression (Primary and Secondary)

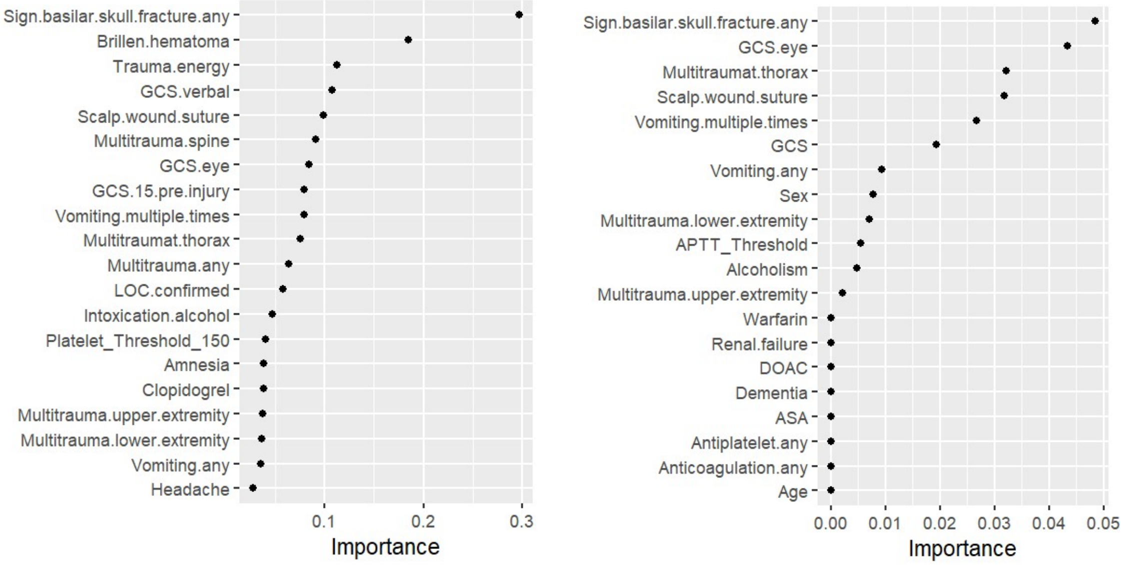

These images demonstrate the topmost 20 important features for Model D on both the Primary (Left) and Secondary (Right) outcomes. The feature importances uses the variance of the overall model described through each variable, noting that the variable to whom the most variance is ascribed to has the most descriptive capacity of the outcome. GCS = Glasgow Coma Scale, LOC = location, S100B = blood biomarker.

Figure B4. Top 20 Features from Model E

General Linear Models (Primary and Secondary)

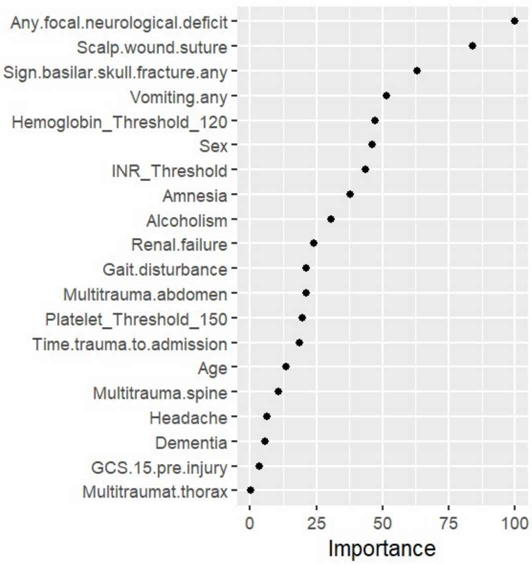

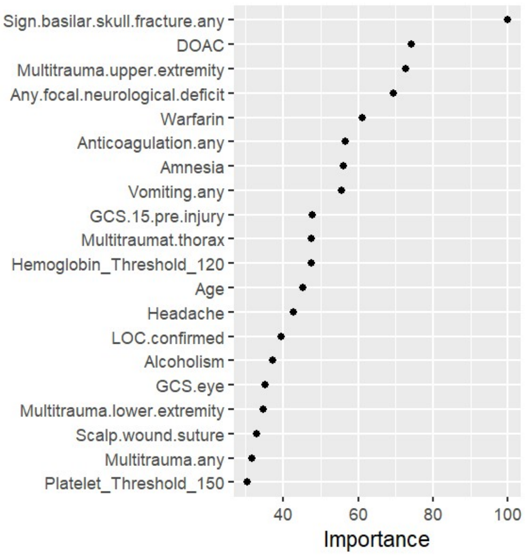

Random Forest (Primary and Secondary)

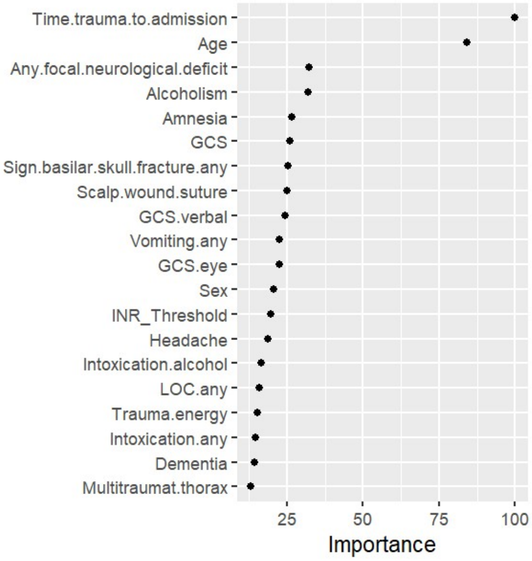

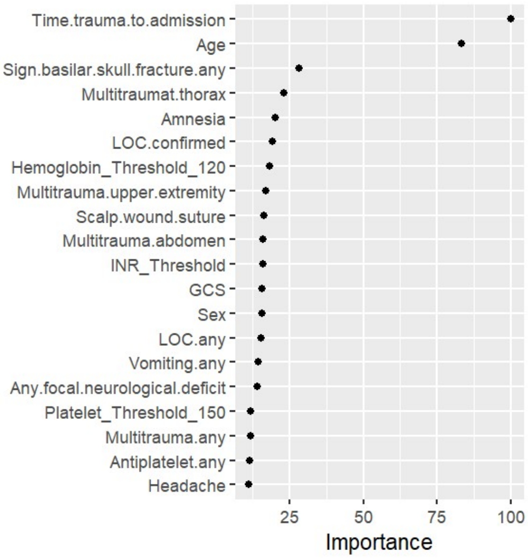

Lasso Regression (Primary and Secondary)

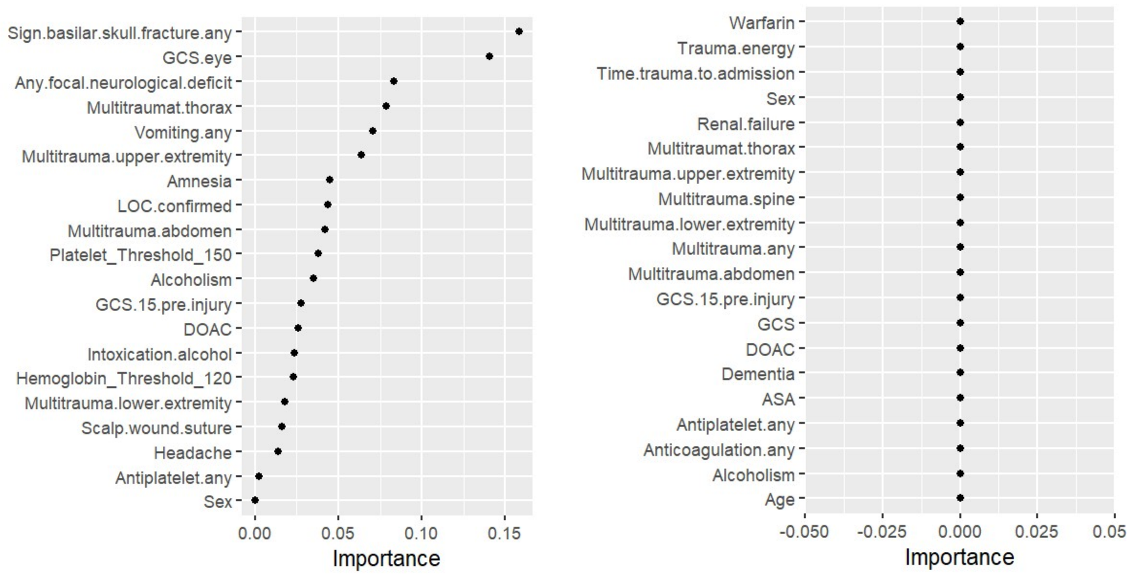

These images demonstrate the topmost 20 important features for Model e on both the Primary (Left) and Secondary (Right) outcomes. The feature importances uses the variance of the overall model described through each variable, noting that the variable to whom the most variance is ascribed to has the most descriptive capacity of the outcome. GCS = Glasgow Coma Scale, LOC = location, S100B = blood biomarker.

Figure B5. Top 20 Features from Model F

General Linear Models (Primary only)

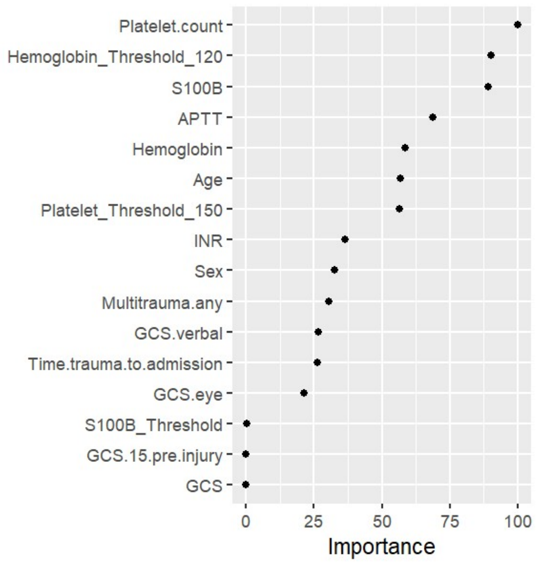

Random Forest (Primary only)

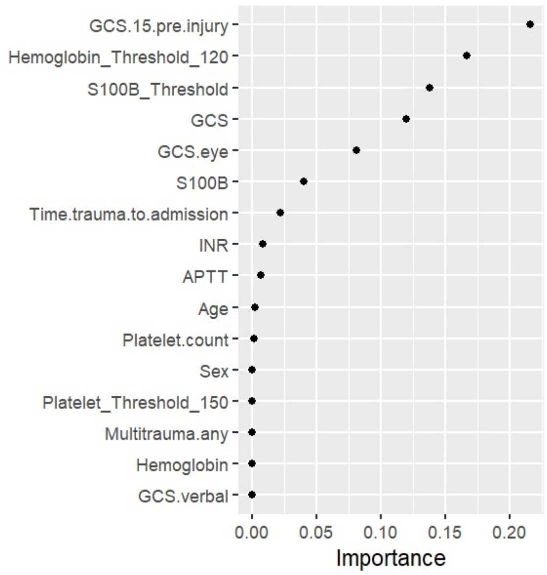

Lasso Regression (Primary only)

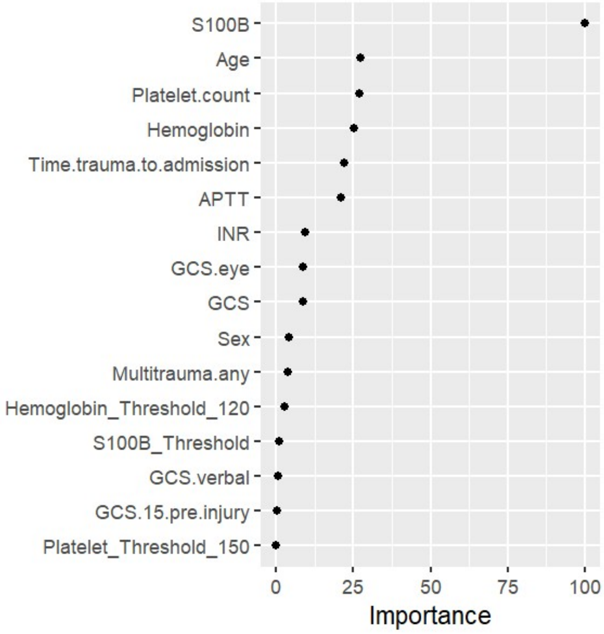

These images demonstrate the topmost 20 important features for Model F on both the Primary outcomes. The feature importances uses the variance of the overall model described through each variable, noting that the variable to whom the most variance is ascribed to has the most descriptive capacity of the outcome. GCS = Glasgow Coma Scale, LOC = location, S100B = blood biomarker.

Appendix C: AUC ROC Curves

Figure C1. AUC ROC curves for Model A

General Linear Models (Primary and Secondary)

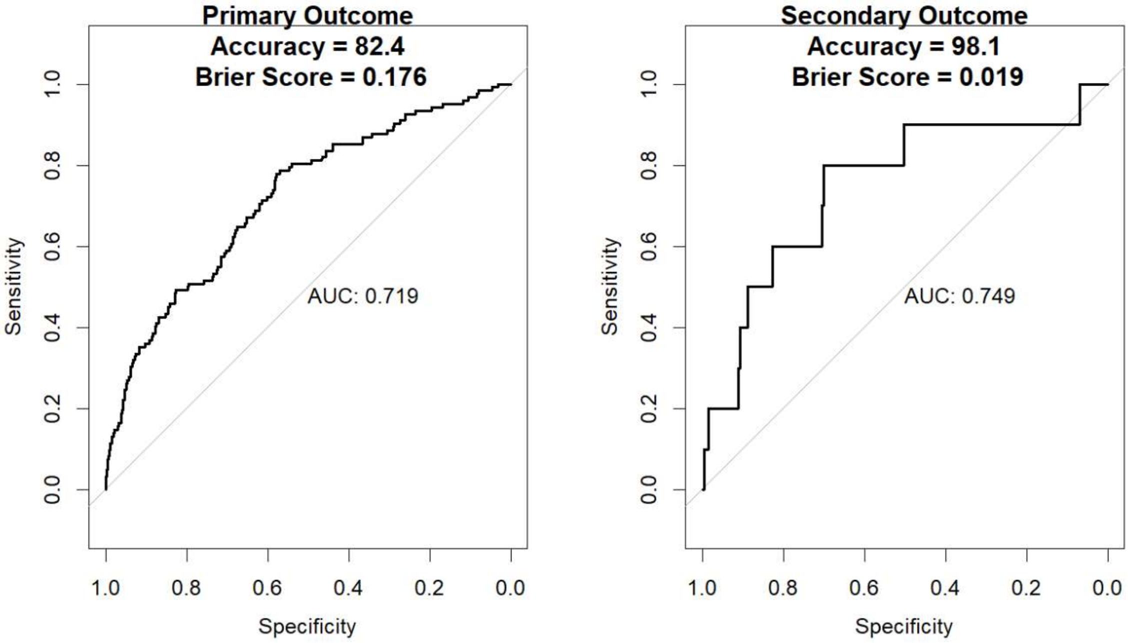

Random Forest (Primary and Secondary)

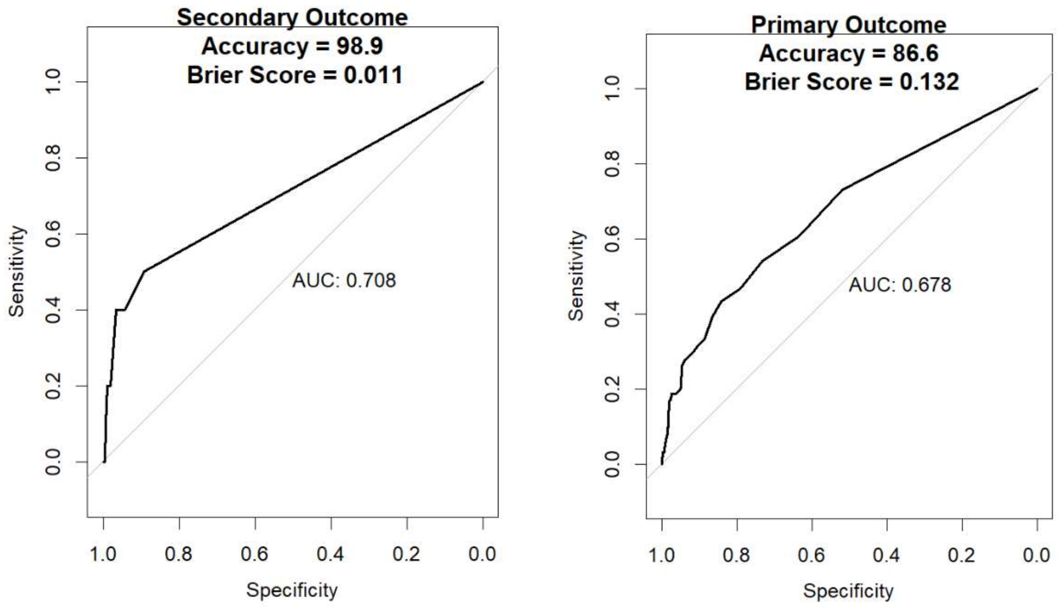

Lasso Regression (Primary and Secondary)

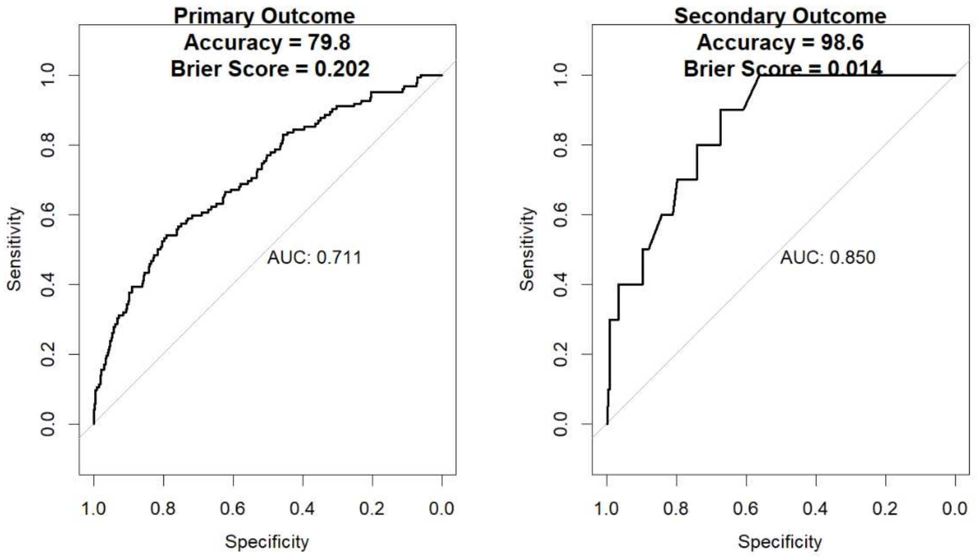

The AUC curves for the Primary (Left) and Secondary (Right) outcomes were found by using the training data to create the model and testing to compare the final results. AUC = area under the curve, ROC = receive operation curve.

Figure C2. AUC ROC curves for Model B

General Linear Models (Primary and Secondary)

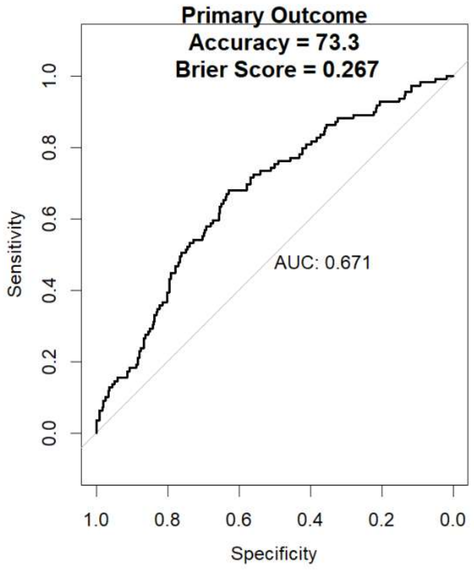

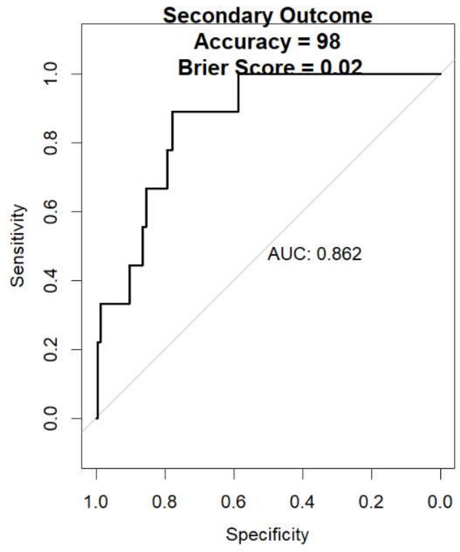

Random Forest (Primary and Secondary)

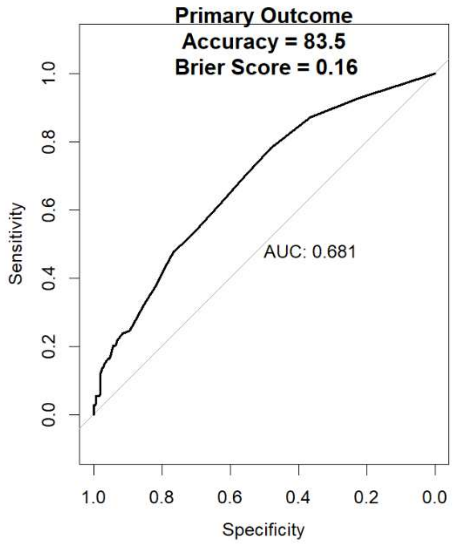

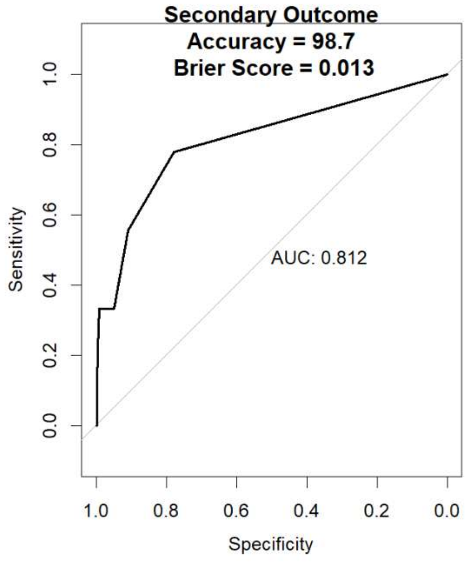

Lasso Regression (Primary and Secondary)

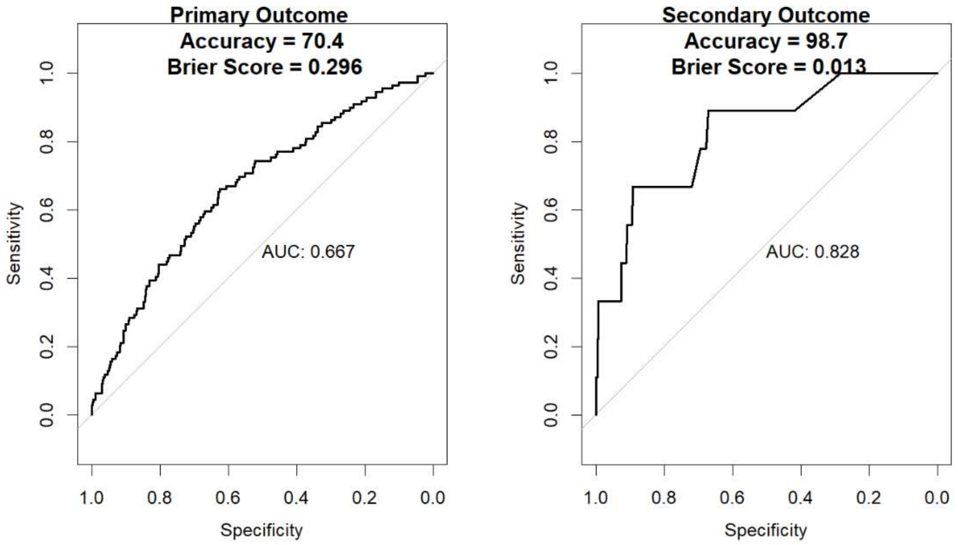

The AUC curves for the Primary (Left) and Secondary (Right) outcomes were found by using the training data to create the model and testing to compare the final results. AUC = area under the curve, ROC = receive operation curve.

Figure C3. AUC ROC curves for Model D

General Linear Models (Primary and Secondary)

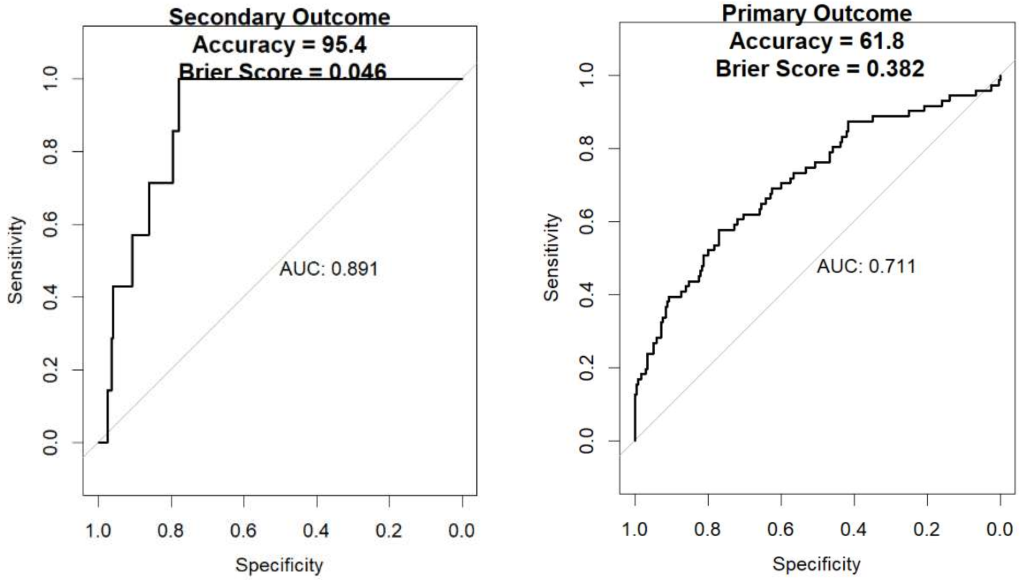

Random Forest (Primary and Secondary)

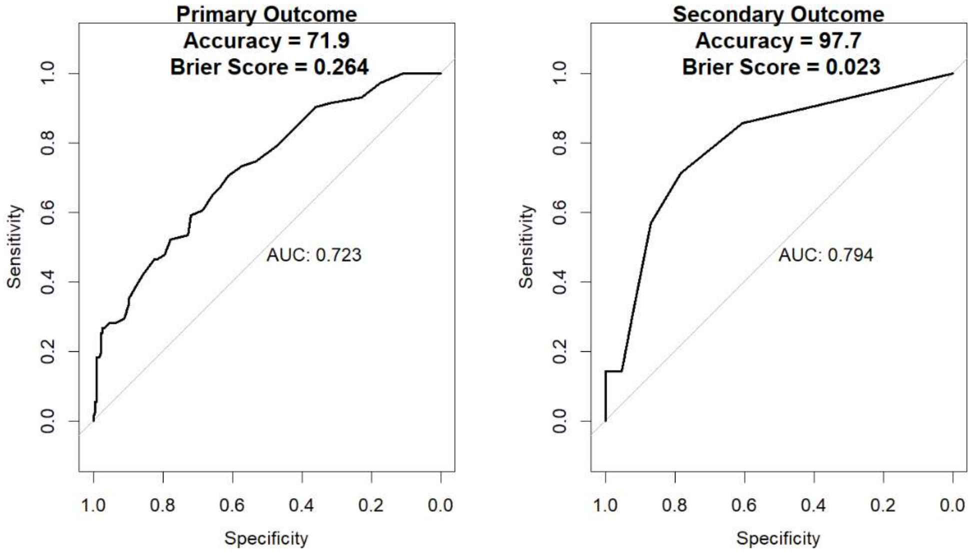

Lasso Regression (Primary and Secondary)

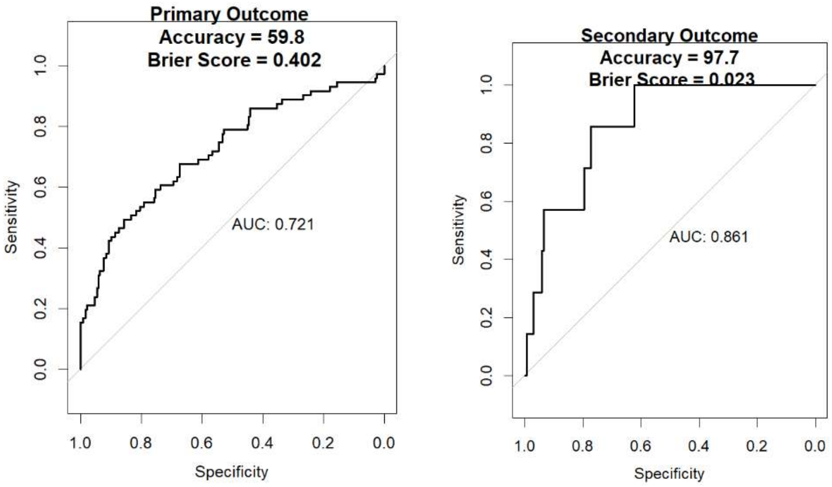

The AUC curves for the Primary (Left) and Secondary (Right) outcomes were found by using the training data to create the model and testing to compare the final results. AUC = area under the curve, ROC = receive operation curve.

Figure C4. AUC ROC curves for Model E

General Linear Models (Primary and Secondary)

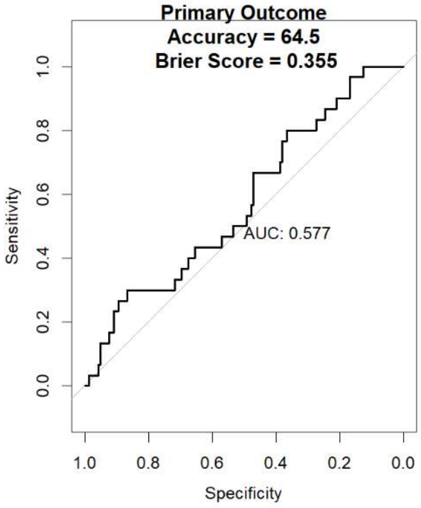

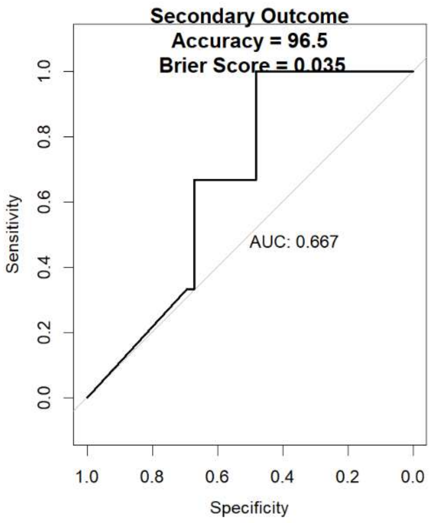

Random Forest (Primary and Secondary)

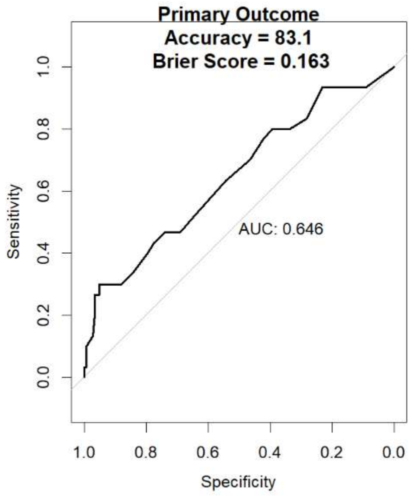

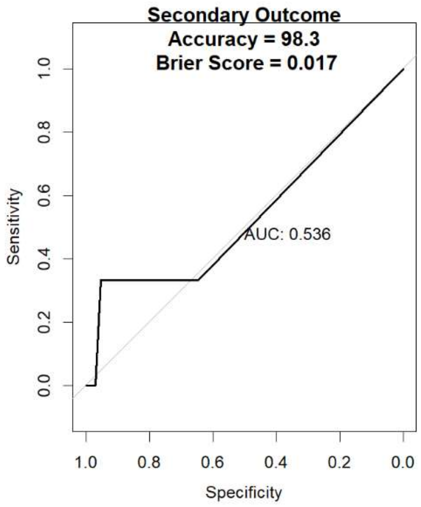

Lasso Regression (Primary and Secondary)

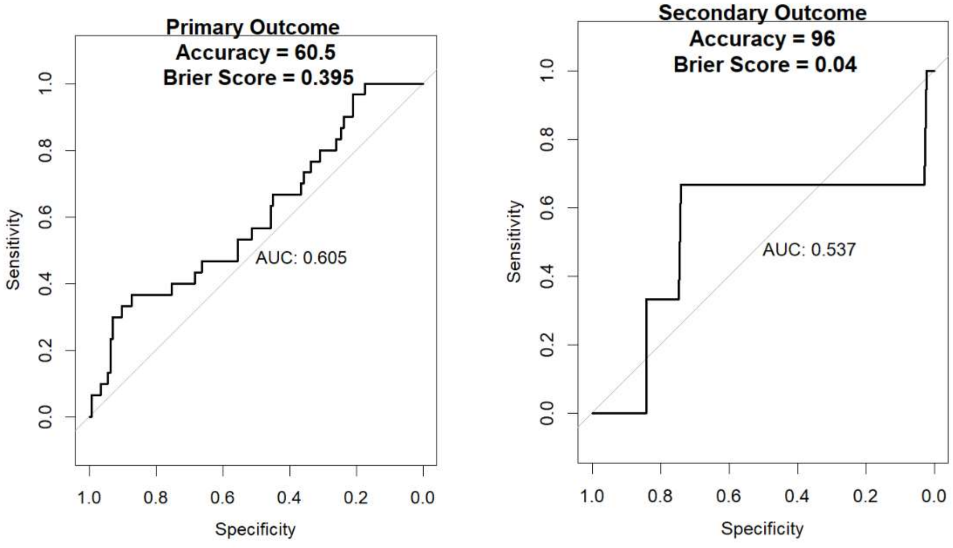

The AUC curves for the Primary (Left) and Secondary (Right) outcomes were found by using the training data to create the model and testing to compare the final results. AUC = area under the curve, ROC = receive operation curve.

Figure C5. AUC ROC curves for Model F

General Linear Models (Primary)

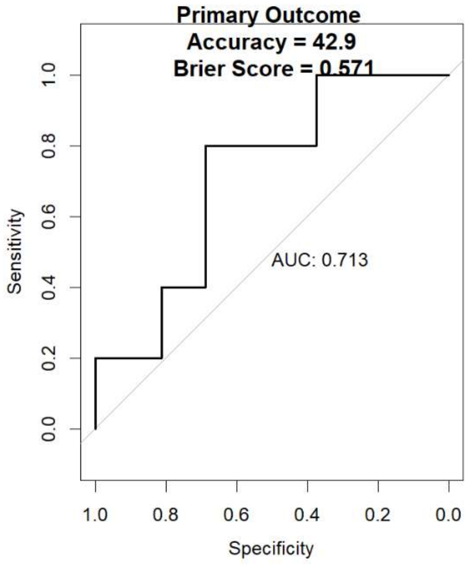

Random Forest (Primary)

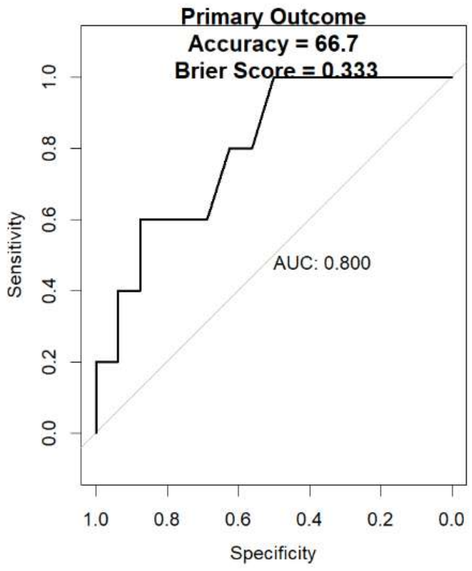

Lasso Regression (Primary)

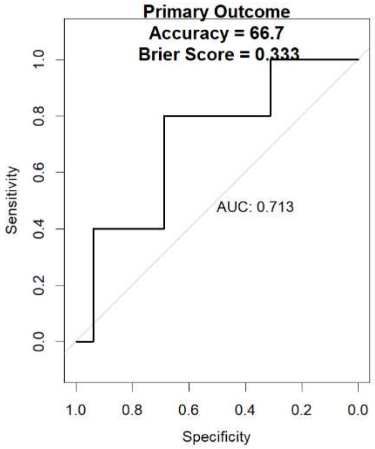

The AUC curves for the Primary outcomes were found by using the training data to create the model and testing to compare the final results. AUC = area under the curve, ROC = receive operation curve.

Appendix D: Outcome Intracranial Hemorrhage

Figure D1. AUC ROC Curve and Top 20 Features for Model A

General Linear Models (Intracranial Hemorrhage Outcome)

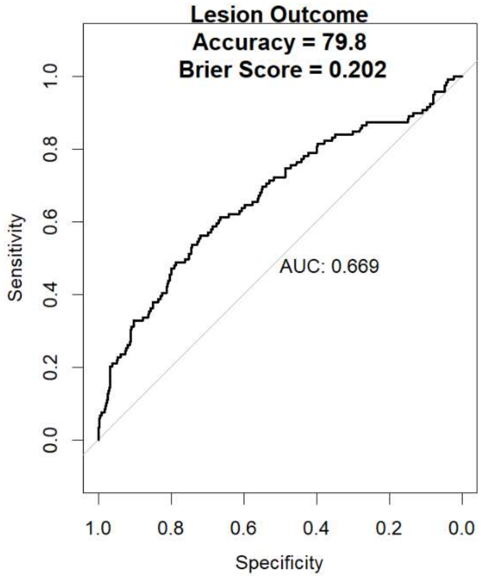

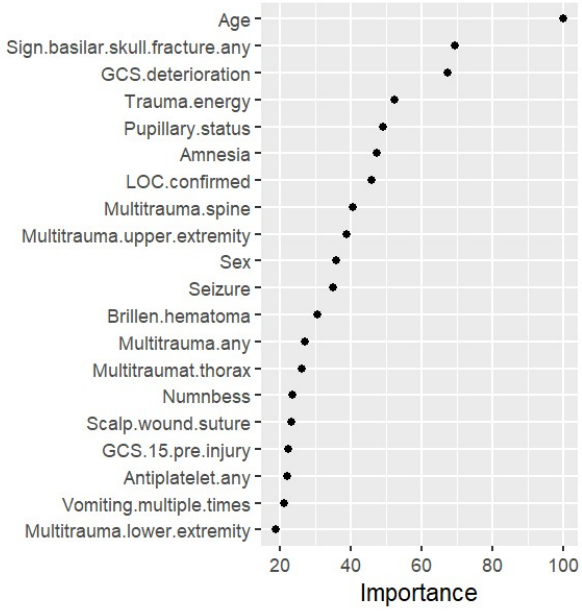

Random Forest (Intracranial Hemorrhage Outcome)

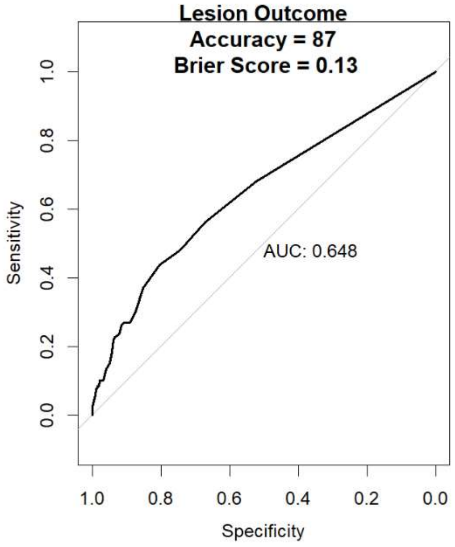

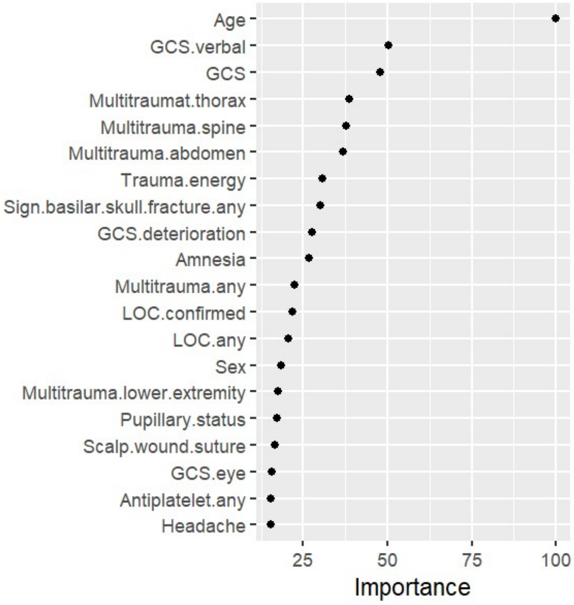

Lasso Regression (Intracranial Hemorrhage Outcome)

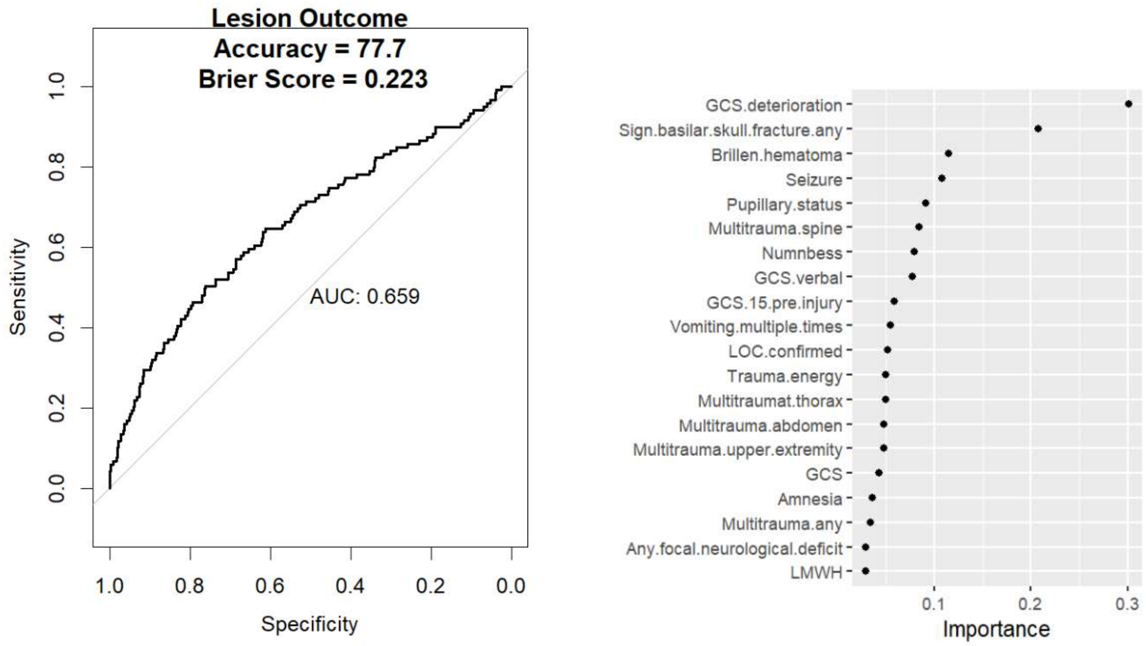

The figure on the left is the AUC curve for the intracranial hemorrhage outcome and the right is the topmost 20 important features for Model A. AUC = area under the curve, GCS = Glasgow Coma Scale, LOC = location, ROC = receive operation curve, S100B = blood biomarker.

Figure D2. AUC ROC Curve and Top 20 Features for Model B

General Linear Models (Intracranial Hemorrhage Outcome)

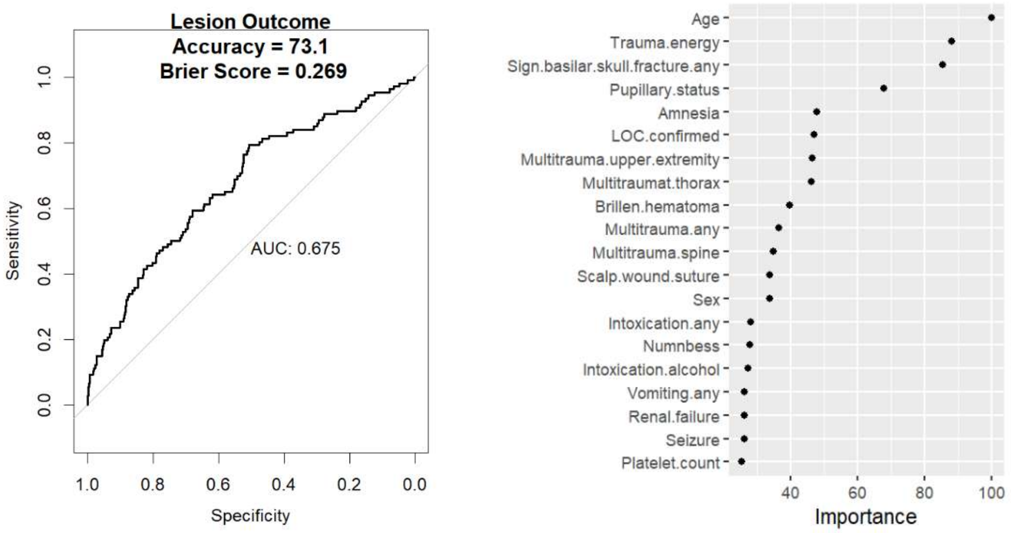

Random Forest (Intracranial Hemorrhage Outcome)

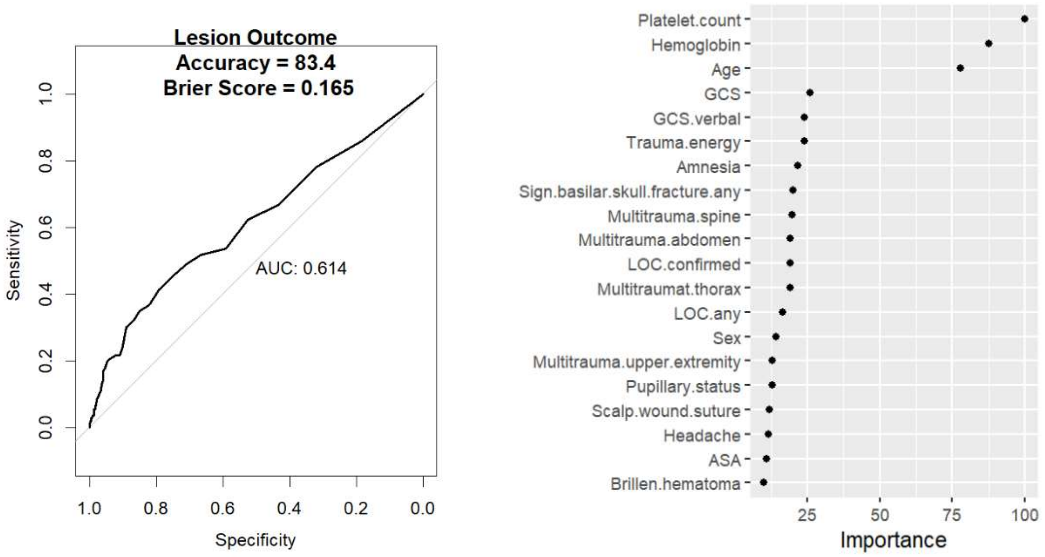

Lasso Regression (Intracranial Hemorrhage Outcome)

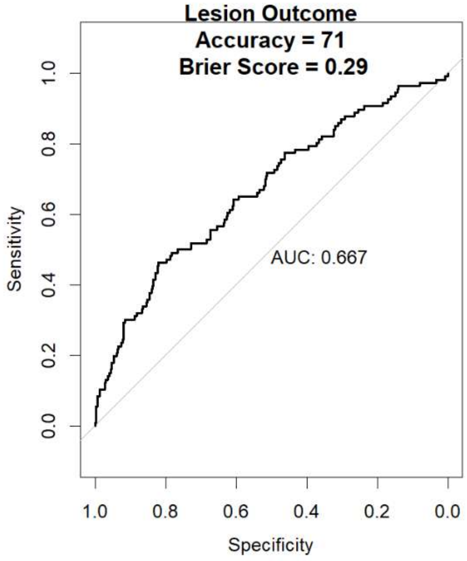

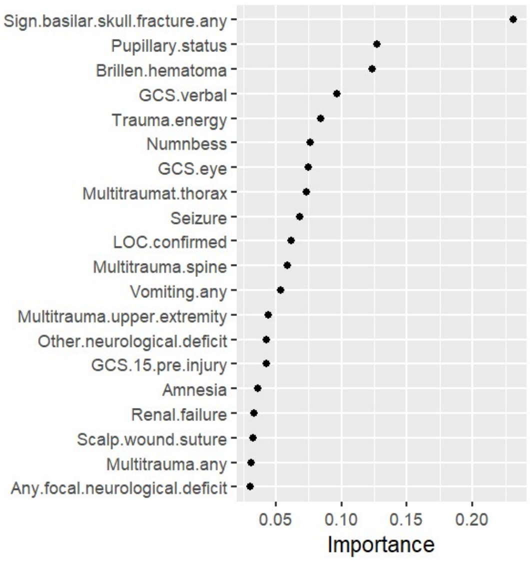

The figure on the left is the AUC curve for the intracranial hemorrhage outcome and the right is the topmost 20 important features for Model B. AUC = area under the curve, GCS = Glasgow Coma Scale, LOC = location, ROC = receive operation curve, S100B = blood biomarker.

Figure D3. AUC ROC Curve and Top 20 Features for Model C

General Linear Models (Intracranial Hemorrhage Outcome)

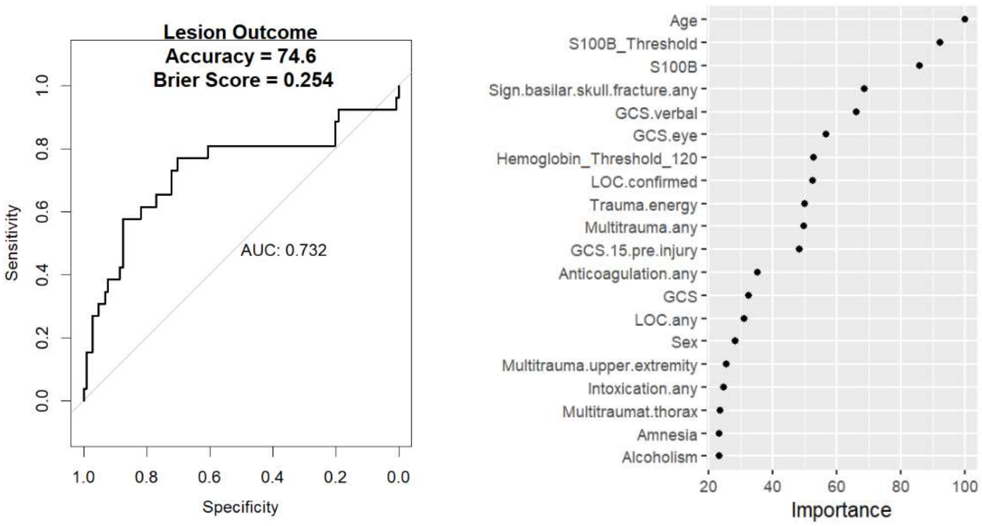

Random Forest (Intracranial Hemorrhage Outcome)

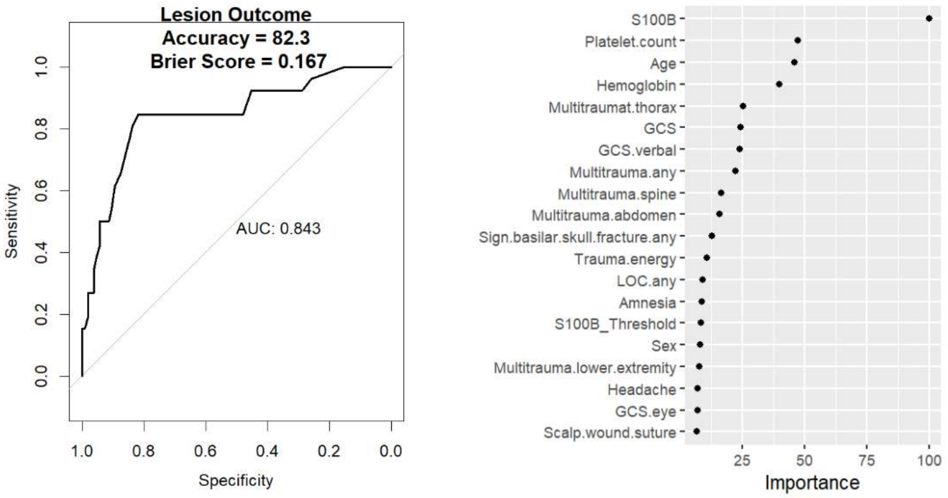

Lasso Regression (Intracranial Hemorrhage Outcome)

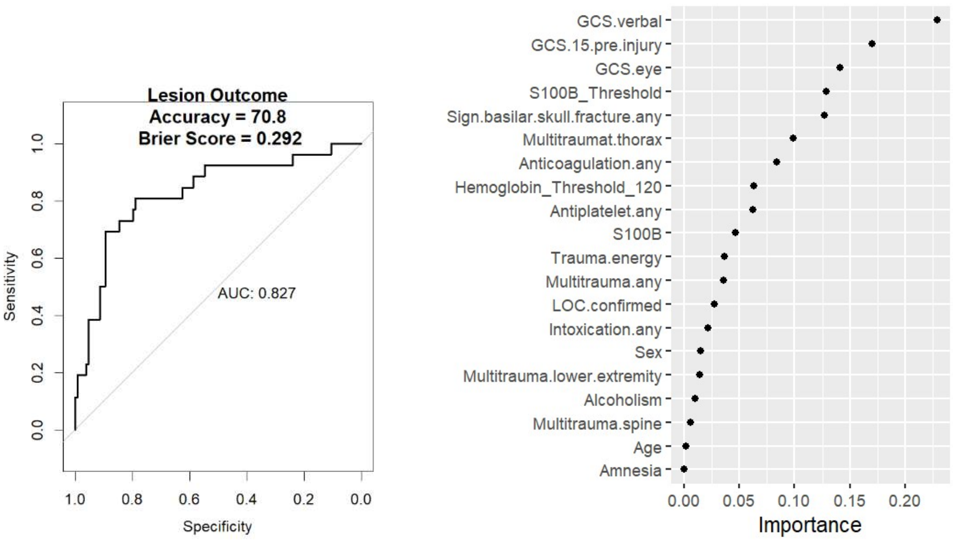

The figure on the left is the AUC curve for the intracranial hemorrhage outcome and the right is the topmost 20 important features for Model C. AUC = area under the curve, GCS = Glasgow Coma Scale, LOC = location, ROC = receive operation curve, S100B = blood biomarker.

Figure D4. AUC ROC Curve and Top 20 Features for Model D

General Linear Models (Intracranial Hemorrhage Outcome)

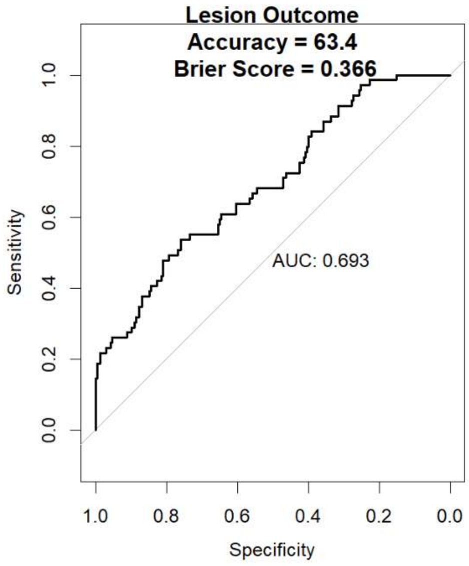

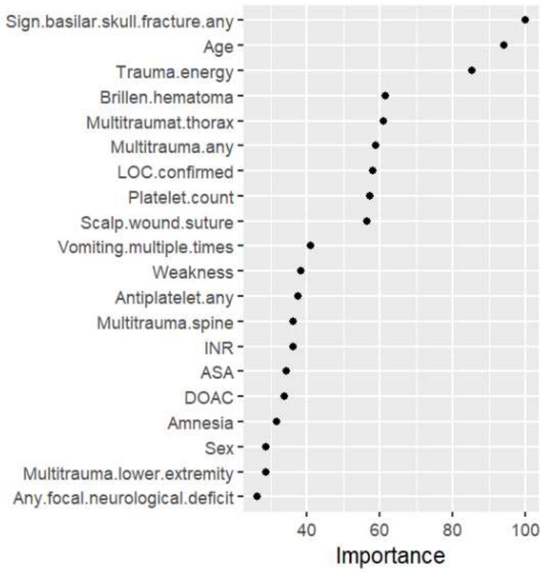

Random Forest (Intracranial Hemorrhage Outcome)

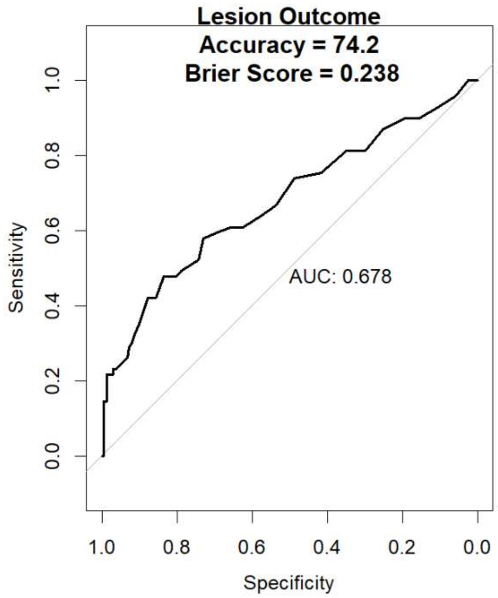

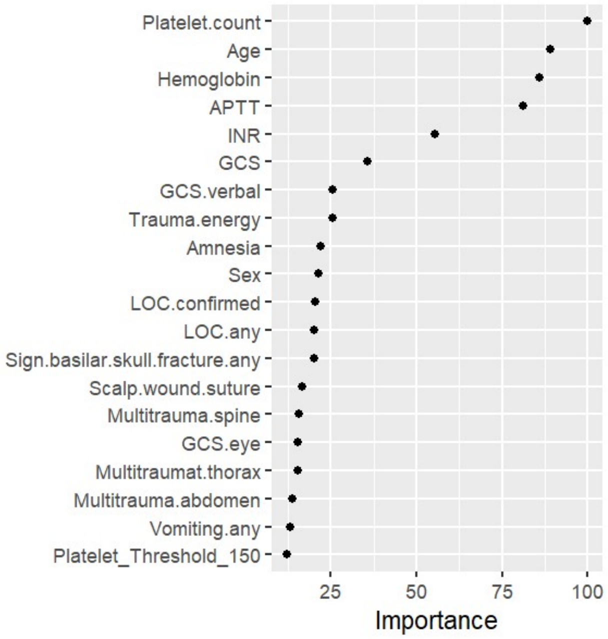

Lasso Regression (Intracranial Hemorrhage Outcome)

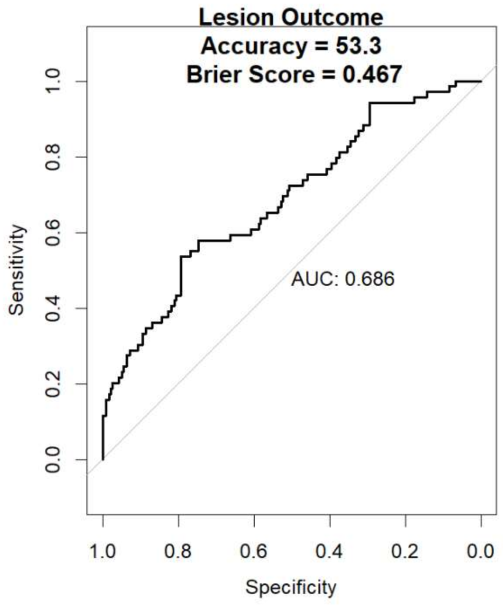

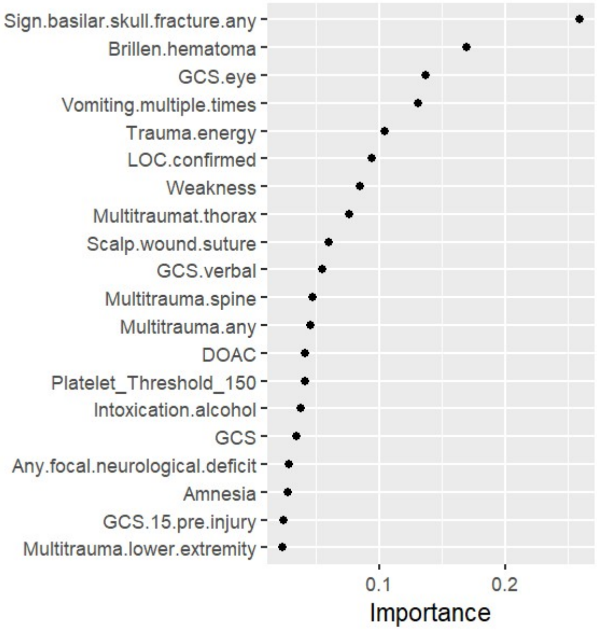

The figure on the left is the AUC curve for the intracranial hemorrhage outcome and the right is the topmost 20 important features for Model D. AUC = area under the curve, GCS = Glasgow Coma Scale, LOC = location, ROC = receive operation curve, S100B = blood biomarker.

Figure D5. AUC ROC Curve and Top 20 Features for Model E

General Linear Models (Intracranial Hemorrhage Outcome)

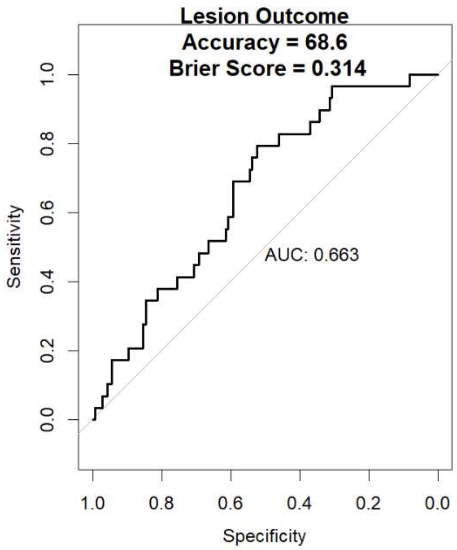

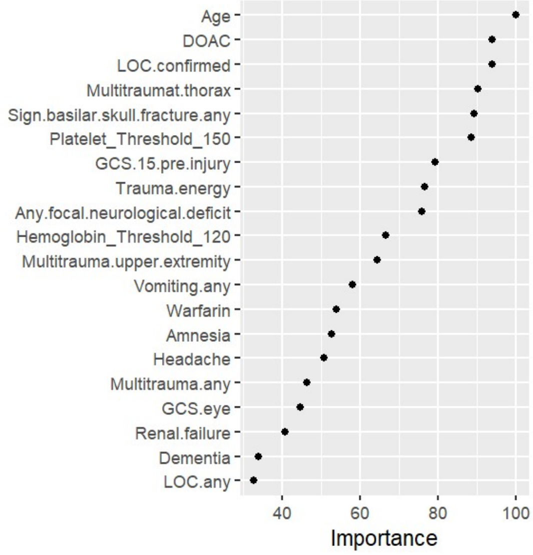

Random Forest (Intracranial Hemorrhage Outcome)

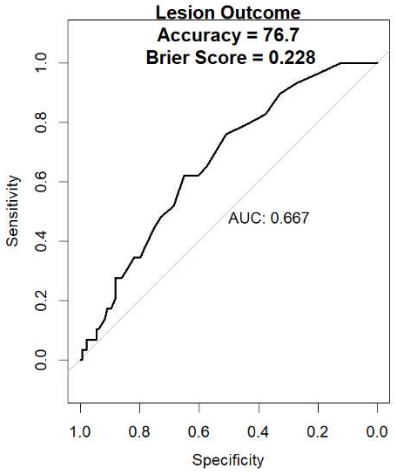

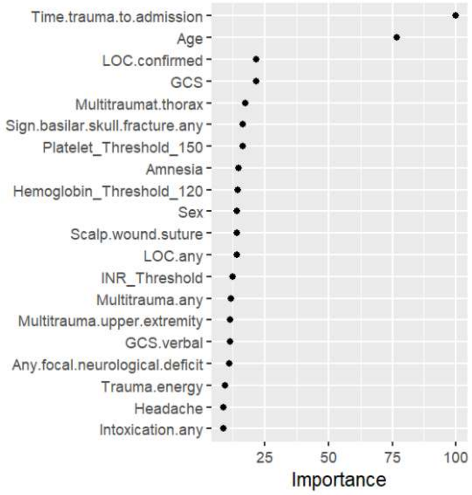

Lasso Regression (Intracranial Hemorrhage Outcome)

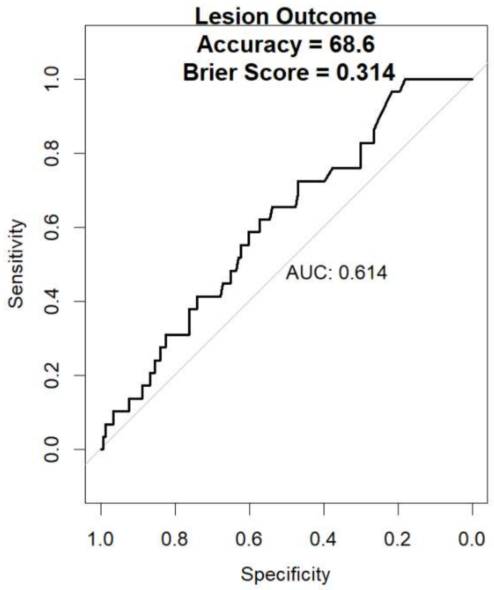

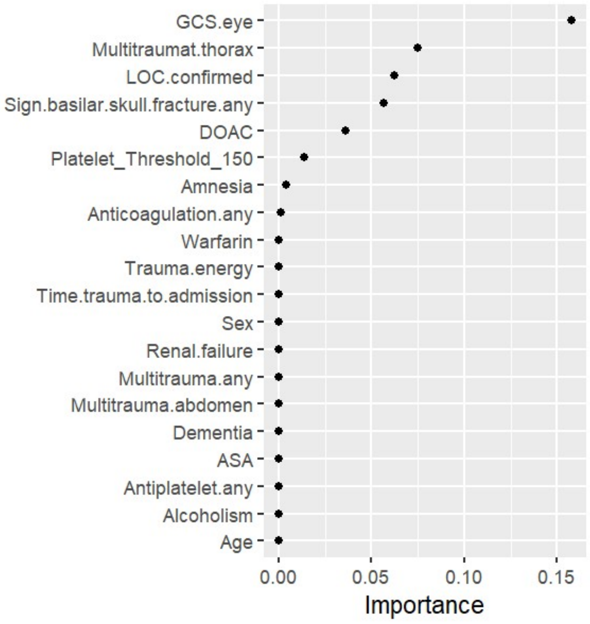

The figure on the left is the AUC curve for the intracranial hemorrhage outcome and the right is the topmost 20 important features for Model E. AUC = area under the curve, GCS = Glasgow Coma Scale, LOC = location, ROC = receive operation curve, S100B = blood biomarker.

Figure D6. AUC ROC Curve and Top 20 Features for Model F

General Linear Models (Intracranial Hemorrhage Outcome)

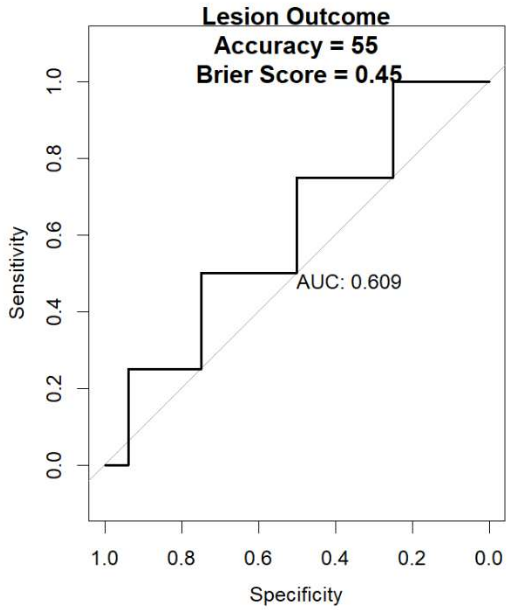

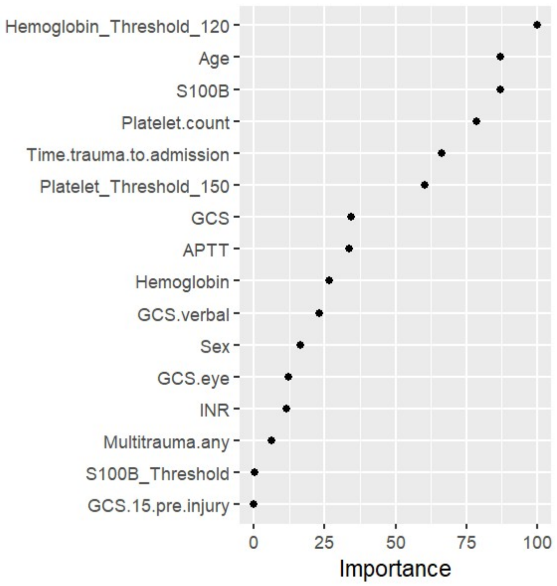

Random Forest (Intracranial Hemorrhage Outcome)

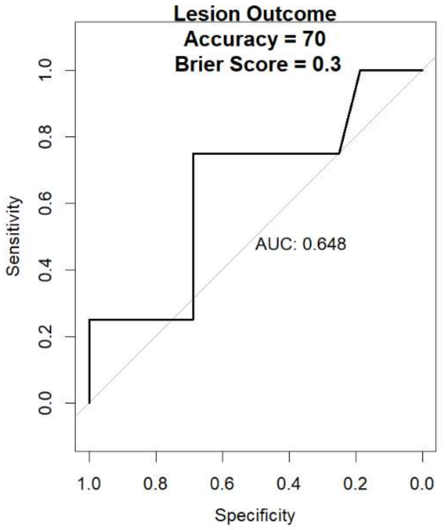

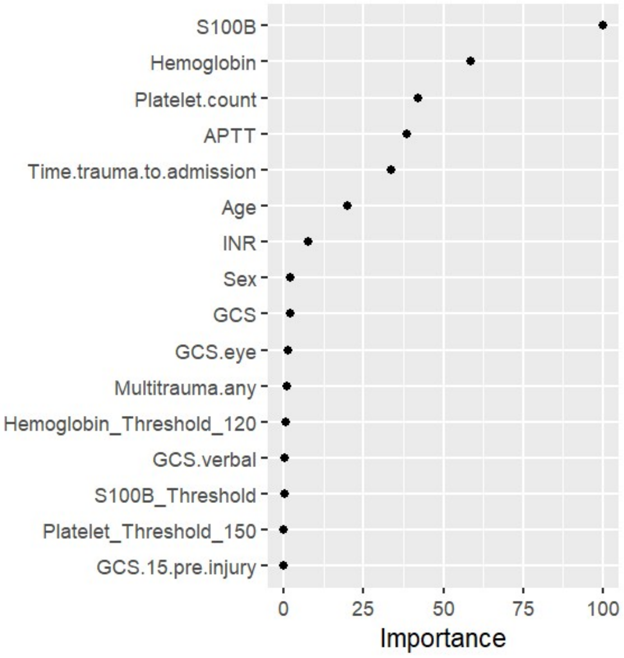

Lasso Regression (Intracranial Hemorrhage Outcome)

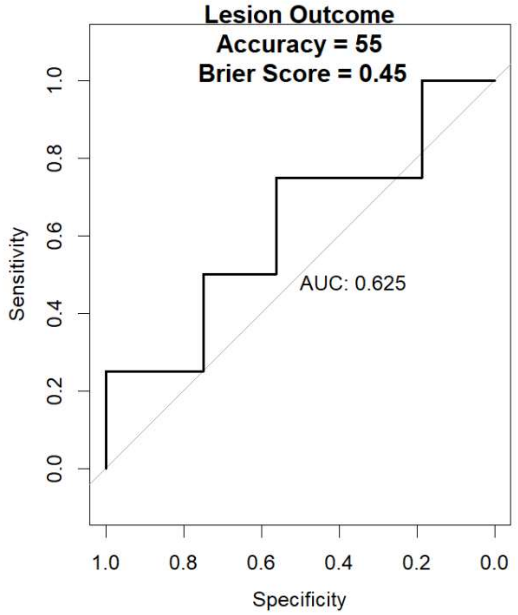

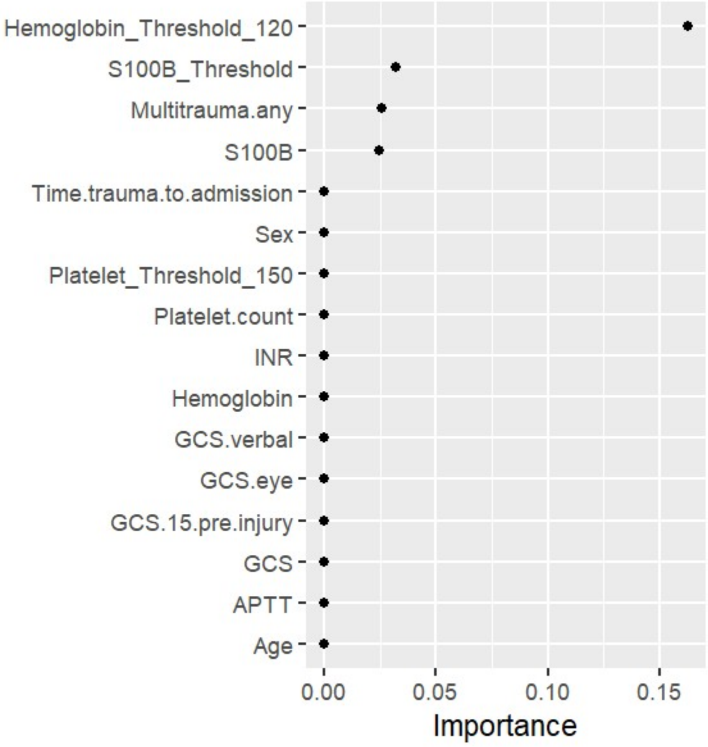

The figure on the left is the AUC curve for the intracranial hemorrhage outcome and the right is the topmost 20 important features for Model F. AUC = area under the curve, GCS = Glasgow Coma Scale, LOC = location, ROC = receive operation curve, S100B = blood biomarker.

Appendix E: Calibration Curves

Figure E1. Calibration curves for Model A

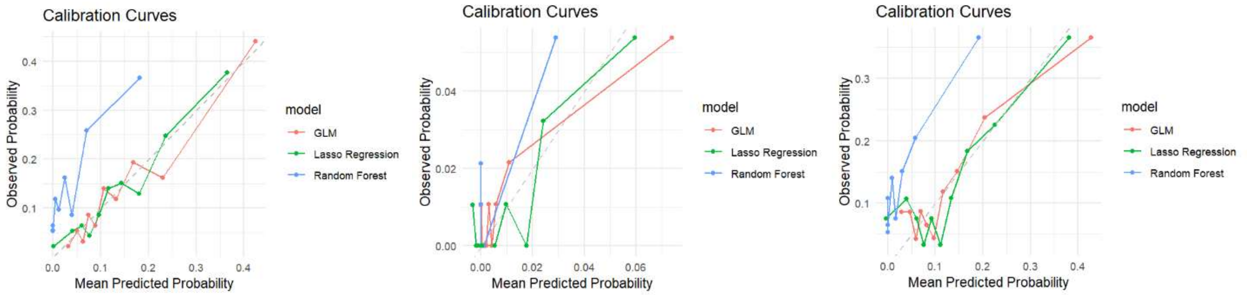

The calibration curves for the Primary (left), Secondary (middle) and Lesion (right) outcomes were found by using the training data to create the model and testing to compare the final results.

Figure E2. Calibration curves for Model B

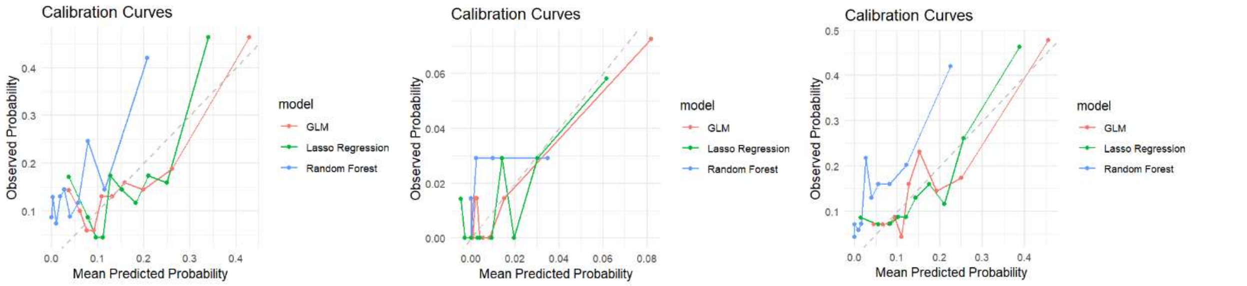

The calibration curves for the Primary (left), Secondary (middle) and Lesion (right) outcomes were found by using the training data to create the model and testing to compare the final results.

Figure E3. Calibration curves for Model C

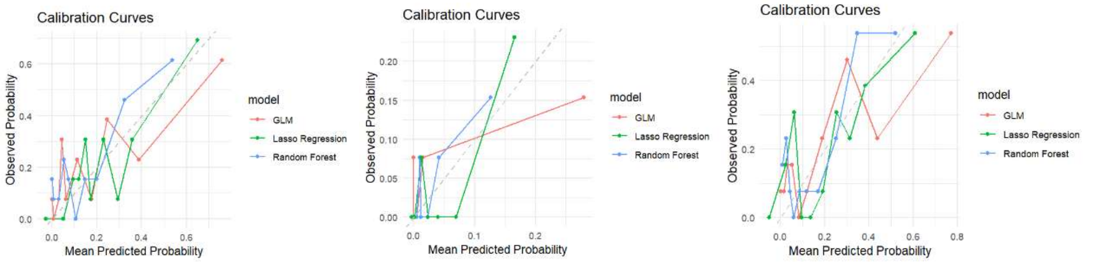

The calibration curves for the Primary (left), Secondary (middle) and Lesion (right) outcomes were found by using the training data to create the model and testing to compare the final results.

Figure E4. Calibration curves for Model D

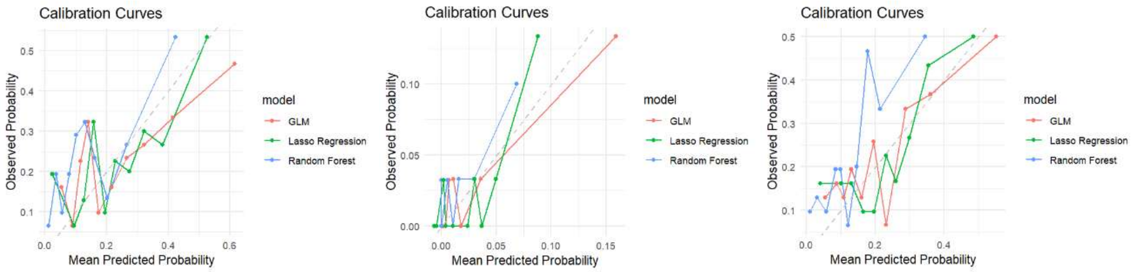

The calibration curves for the Primary (left), Secondary (middle) and Lesion (right) outcomes were found by using the training data to create the model and testing to compare the final results.

Figure E5. Calibration curves for Model E

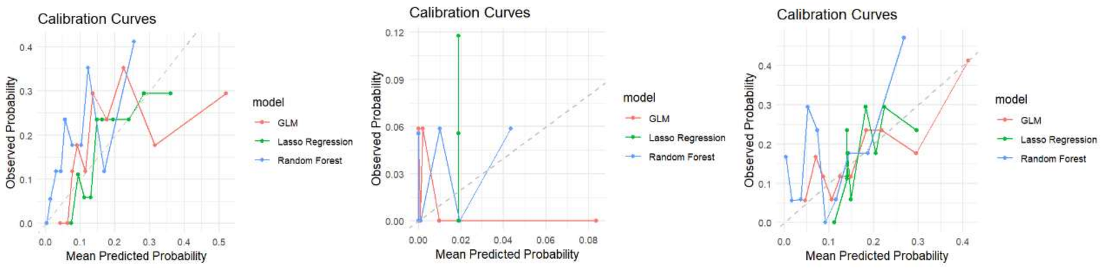

The calibration curves for the Primary (left), Secondary (middle) and Lesion (right) outcomes were found by using the training data to create the model and testing to compare the final results.

Figure E6. Calibration curves for Model F

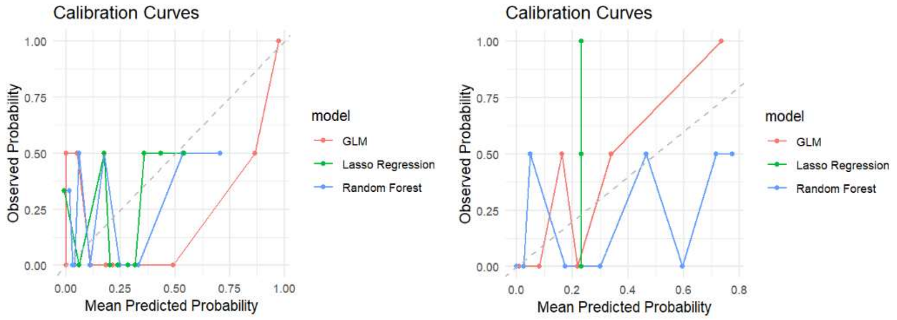

The calibration curves for the Primary (left), and Lesion (right) outcomes were found by using the training data to create the model and testing to compare the final results.

Appendix F: VIF for GLM

r me	Model_A_Primary	Model_A_Secondary	Model_A_Lesion	Model_B_Primary	Model_B_Secondary	Model_B_Lesion	Model_C_Primary	Model_C_Secondary	Model_C_Lesion	Model_D_Primary	Model_D_Secondary	Model_D_Lesion	Model_E_Primary	Model_E_Secondary	Model_E_Lesion	Model_F_Primary
Age	2.23	2.51	2.23	2.32	2.41	2.35	2.21	2.38	2.19	2.34	2.51	2.19	2.42	3.58	2.60	2.03
Alcoholism	1.47	1.54	1.43	1.38	1.45	1.40	1.25	1.84	1.24	1.41	1.60	1.41	1.49	2.75	1.40	
Amnesia	1.13	1.21	1.14	1.15	1.30	1.13	1.17	1.38	1.15	1.14	1.47	1.13	1.22	2.23	1.22	
Anticoagulation.any	>1000	>1000	>1000	>1000	>1000	>1000	1.32	1.73	1.38	7.57	5.91	8.01	6.51	4.08	8.11	
Antiplatelet.any	47.89	>1000	29.26	23.73	>1000	23.94	8.65	12.45	6.82	19.78	>1000	21.20	5.51	>1000	5.73	
Any.focal.neurological.deficit	2.32	2.29	2.39	2.25	2.93	2.40				1.94	1.76	2.27	1.10	2.92	1.11	
APTT										3.02	3.96	3.10				1.81
APTT_Threshold										2.60	3.17	2.61				
ASA	43.03	>1000	26.44	20.02	>1000	20.42	8.52	12.45	6.74	16.10	>1000	17.03	5.19	>1000	5.46	
Blurry.vision	1.02	1.00	1.02													
Brillen.hematoma	2.99	1.86	2.70	3.27	2.01	3.22				3.04	2.63	2.56				
Clopidogrel	9.76	>1000	6.02	4.74	1.00	4.25				3.93	1.00	5.34				
DAPT	2.59	>1000	1.82													
Dementia	2.05	1.97	2.04	2.06	3.02	2.10				2.19	3.53	2.08	1.83	2.04	2.04	
DOAC	>1000	>1000	>1000	>1000	>1000	>1000				5.20	3.27	5.54	4.20	1.00	5.18	
Gait.disturbance	1.09	1.16	1.10	1.08	1.17	1.09				1.07	1.33	1.08	1.11	2.77	1.10	
GCS	29.93	19.48	33.36	50.08	17.49	38.99	41.14	20.40	>1000	54.21	27.85	55.51	45.46	>1000	35.79	>1000
GCS.15.pre.injury	2.14	2.04	2.13	2.12	3.11	2.19	1.13	1.00	1.11	2.16	3.17	2.05	1.85	2.55	2.05	1.00
GCS.deterioration	1.04	1.27	1.04													
GCS.eye	6.13	6.09	6.53	9.59	4.98	8.05	11.62	7.75	>1000	10.15	8.14	11.56	6.83	>1000	8.17	>1000
GCS.verbal	21.71	11.55	24.39	36.60	11.43	27.90	28.14	12.58	>1000	41.19	18.81	39.58	35.43	>1000	27.80	>1000
Headache	1.17	1.37	1.17	1.13	1.27	1.13	1.10	1.48	1.14	1.17	1.35	1.11	1.26	2.07	1.31	
Hemoglobin				2.41	2.68	2.45	2.28	2.73	2.21	2.47	1.40	2.42				7.23

Hemoglobin_Threshold_120				2.18	2.51	2.20	2.16	2.58	2.02	2.16	1.62	2.16	1.19	1.61	1.24	10.55
INR										3.73	5.42	3.90				1.50
INR_Threshold													1.43	2.21	1.53	
Intoxication.alcohol	15.80	>1000	15.97	19.00	8.32	13.75	28.86	>1000	>1000	17.30	>1000	16.36	32.37	880459122.77	15.53	
Intoxication.any	15.89	>1000	16.05	19.13	8.67	13.77	28.75	>1000	>1000	17.17	>1000	16.21	32.31	880459118.39	15.55	
LMWH	>1000	>1000	>1000	>1000	>1000	>1000										
LOC.any	2.37	4.22	2.49	2.47	4.70	2.42	2.97	5.09	3.11	2.87	3.87	2.53	3.33	>1000	3.49	
LOC.confirmed	2.35	4.16	2.48	2.43	4.76	2.41	2.93	4.47	3.05	2.83	3.70	2.48	3.27	>1000	3.69	
Multitrauma.abdomen	4.82	5.60	4.71	4.61	9.45	4.76	9.24	32.94	10.23	5.91	7.61	6.22	3.97	>1000	3.75	
Multitrauma.any	3.69	6.86	3.80	3.70	>1000	3.67	3.79	>1000	3.79	4.03	>1000	4.04	3.89	>1000	3.88	1.90
Multitrauma.lower.extremity	1.37	1.32	1.35	1.37	1.49	1.38	1.48	1.75	1.49	1.40	1.33	1.39	1.59	3.07	1.50	
Multitrauma.spine	2.97	3.81	2.82	2.94	6.79	3.07	4.99	6.02	6.90	3.64	4.69	3.92	2.62	>1000	2.66	
Multitrauma.upper.extremity	1.65	1.45	1.70	1.61	1.60	1.63	1.71	1.84	1.77	1.50	1.53	1.53	2.00	1.00	1.86	
Multitraumat.thorax	4.55	8.38	4.59	4.44	>1000	4.40	7.03	>1000	7.64	5.96	>1000	6.20	3.57	>1000	3.63	
Numnbess	1.33	1.00	1.36	1.35	1.00	1.38										
Other.neurological.deficit	1.05	1.12	1.05	1.05	1.37	1.05										
Platelet.count				1.36	1.37	1.45	1.29	2.45	1.30	1.63	1.87	1.65				2.34
Platelet_Threshold_150				1.35	1.30	1.43	1.28	2.22	1.33	1.60	1.80	1.60	1.16	1.62	1.10	1.63
Pupillary.status	1.04	1.13	1.05	1.03	1.05	1.05										
Re l.failure	1.07	1.12	1.07	1.08	1.17	1.10				1.12	1.41	1.12	1.18	1.91	1.18	
S100B							1.19	1.68	1.19							1.21
S100B_Threshold							1.06	1.00	1.04							1.00

Scalp.wound.suture	1.04	1.13	1.04	1.04	1.13	1.04	1.11	1.93	1.09	1.06	1.30	1.06	1.10	2.14	1.13	
Seizure	1.07	1.35	1.06	1.05	1.24	1.07										
Sex	1.15	1.13	1.16	1.22	1.27	1.24	1.38	1.89	1.40	1.26	1.34	1.24	1.25	1.51	1.29	1.63
Sign.basilar.skull.fracture.any	2.98	1.88	2.70	3.27	2.12	3.20	1.14	2.16	1.11	3.05	2.79	2.59	1.10	2.26	1.14	
Time.trauma.to.admission													1.23	3.15	1.19	2.15
Trauma.energy	1.83	2.00	1.78	1.89	2.15	1.88	2.28	2.15	2.26	2.11	2.36	2.14	2.02	2.62	1.98	
Vomiting.any	2.37	2.09	2.59	2.39	2.10	2.40				2.52	2.70	2.52	1.23	1.00	1.20	
Vomiting.multiple.times	2.38	2.08	2.56	2.39	1.98	2.40				2.48	2.57	2.45				
Warfarin	>1000	>1000	>1000	>1000	>1000	>1000				6.45	8.30	6.72	4.72	4.03	5.87	
Weakness	1.87	2.14	1.92	1.82	2.84	1.92				1.91	1.66	2.25				

The variation inflation factor for general linear model.
